# Supplementary material for: Mitochondrial RNase H1 activity regulates R-loop homeostasis to maintain genome integrity and enable early embryogenesis in Arabidopsis
Source: PLoS Biol. 2021 Aug 3;19(8):e3001357. doi: 10.1371/journal.pbio.3001357 (PMC8330923; doi:10.1371/journal.pbio.3001357)
Supplement: S1 Fig — (A) Structure of purified GST-fused proteins. GST tag was fused to the N terminus of AtRNH1B without the MTS (GST-AtRNH1B). The Asp (191 D) was mutated to Asn (N) in GST-AtRNH1B fusion protein (GST-AtRNH1BM). (B) SDS-PAGE of purified recombinant proteins. The molecular weight of GST and GST-AtRNH1B/M are approximately 25 kDa and approximately 63 kDa, respectively. (C) Related to Fig 1D, the amounts of proteins used in Coomassie blue staining are 10 times of purified protein used in RNase H activity assay. (D) The RNA:DNA hybrid substrate with FAM-labeled RNA (100 nM) following 5 minutes of incubation with increasing concentrations of GST-AtRNH1B and GST-AtRNH1BM. (E) Microscopic observation of GUS signals in epidermal and mesophyll cells of AtRNH1Bpro:AtRNH1B-GUS atrnh1b-1 after GUS staning. For comparison, the cells were selected at same area through changing focal length. Col-0 was included as negative control to indicate the cell without GUS activity, which was shown orange-red without blue GUS signals as the contrast under microscopy. Scale bars, 100 μm. The data underlying this figure can be found in S1 Raw Images. GST, glutathione-S-transferase; GUS, β-glucuronidase enzyme; SDS-PAGE, sodium dodecyl sulfate-PAGE. (PPTX) [file pbio.3001357.s001.pptx]

## Slide 1
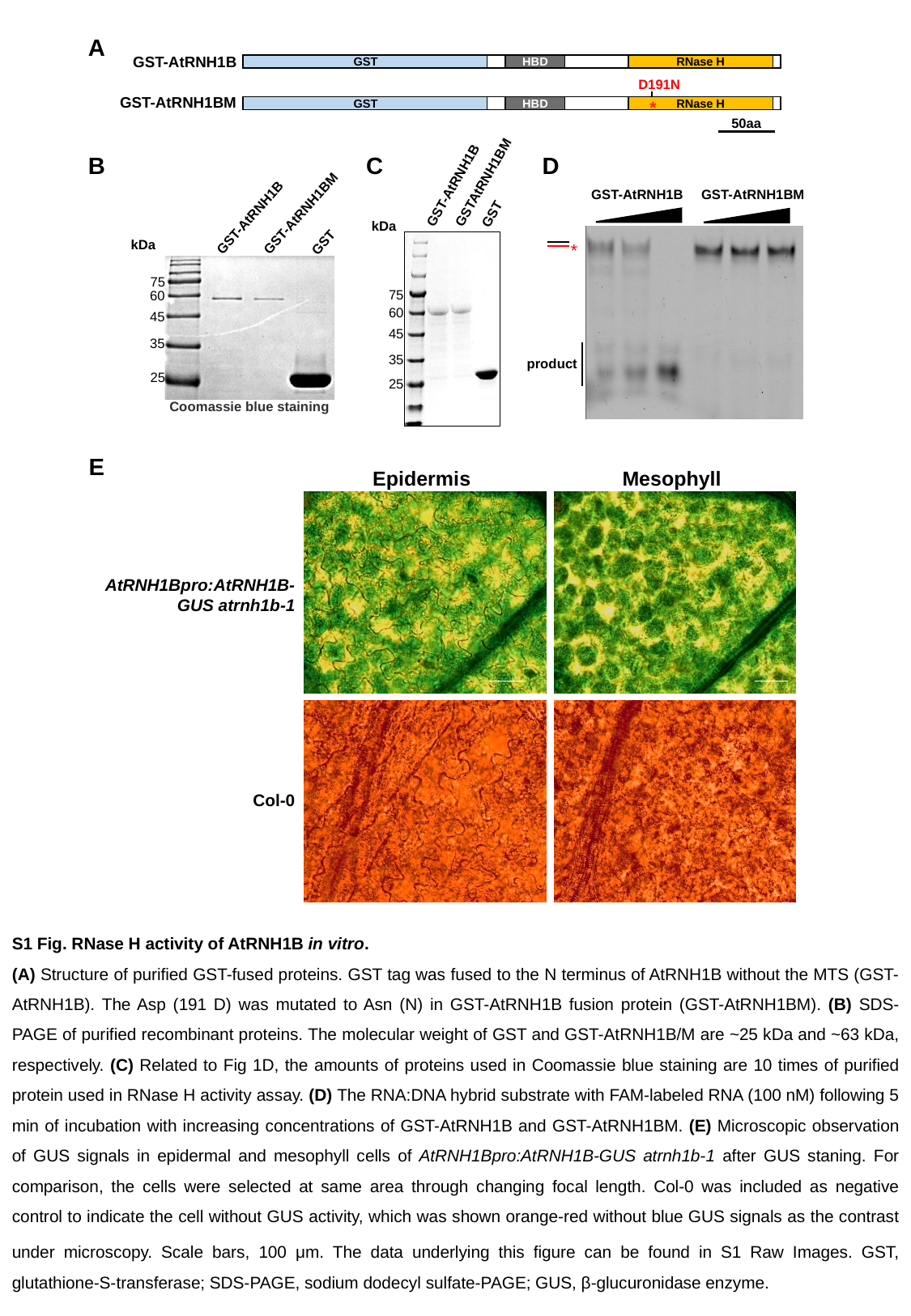

A
GST-AtRNH1B
HBD
RNase H
GST
D191N
*
HBD
RNase H
GST
GST-AtRNH1BM
50aa
GSTAtRNH1BM
GST-AtRNH1B
GST
kDa
75
60
45
35
25
B
C
D
GST-AtRNH1BM
GST-AtRNH1B
GST
kDa
75
60
45
35
25
Coomassie blue staining
GST-AtRNH1B
GST-AtRNH1BM
*
product
E
Epidermis
Mesophyll
AtRNH1Bpro:AtRNH1B-GUS atrnh1b-1
Col-0
S1 Fig. RNase H activity of AtRNH1B in vitro.
(A) Structure of purified GST-fused proteins. GST tag was fused to the N terminus of AtRNH1B without the MTS (GST-AtRNH1B). The Asp (191 D) was mutated to Asn (N) in GST-AtRNH1B fusion protein (GST-AtRNH1BM). (B) SDS-PAGE of purified recombinant proteins. The molecular weight of GST and GST-AtRNH1B/M are ~25 kDa and ~63 kDa, respectively. (C) Related to Fig 1D, the amounts of proteins used in Coomassie blue staining are 10 times of purified protein used in RNase H activity assay. (D) The RNA:DNA hybrid substrate with FAM-labeled RNA (100 nM) following 5 min of incubation with increasing concentrations of GST-AtRNH1B and GST-AtRNH1BM. (E) Microscopic observation of GUS signals in epidermal and mesophyll cells of AtRNH1Bpro:AtRNH1B-GUS atrnh1b-1 after GUS staning. For comparison, the cells were selected at same area through changing focal length. Col-0 was included as negative control to indicate the cell without GUS activity, which was shown orange-red without blue GUS signals as the contrast under microscopy. Scale bars, 100 μm. The data underlying this figure can be found in S1 Raw Images. GST, glutathione-S-transferase; SDS-PAGE, sodium dodecyl sulfate-PAGE; GUS, β-glucuronidase enzyme.
