# Supplementary material for: Mitochondrial RNase H1 activity regulates R-loop homeostasis to maintain genome integrity and enable early embryogenesis in Arabidopsis
Source: PLoS Biol. 2021 Aug 3;19(8):e3001357. doi: 10.1371/journal.pbio.3001357 (PMC8330923; doi:10.1371/journal.pbio.3001357)
Supplement: S2 Fig — (A) Gene structure of AtRNH1B genomic DNA. White boxes represent UTRs, black boxes represent exons, and black lines represent introns. The triangles point to the positions of T-DNA insertions in atrnh1b-1 and atrnh1b-2. (B, C) Twenty-eight cycles of RT-PCR of AtRNH1B transcript in atrnh1b-1 (B) and atrnh1b-2 (C); primers are shown in A. GAPDH was used as the reference gene. (D) Col-0 and atrnh1b plants at the vegetative stage (upper) and reproductive stage (bottom). Scale bars, 1 cm. The data underlying this figure can be found in S1 Raw Images. RT-PCR, reverse transcription PCR; UTR, untranslated region. (PPTX) [file pbio.3001357.s002.pptx]

## Slide 1
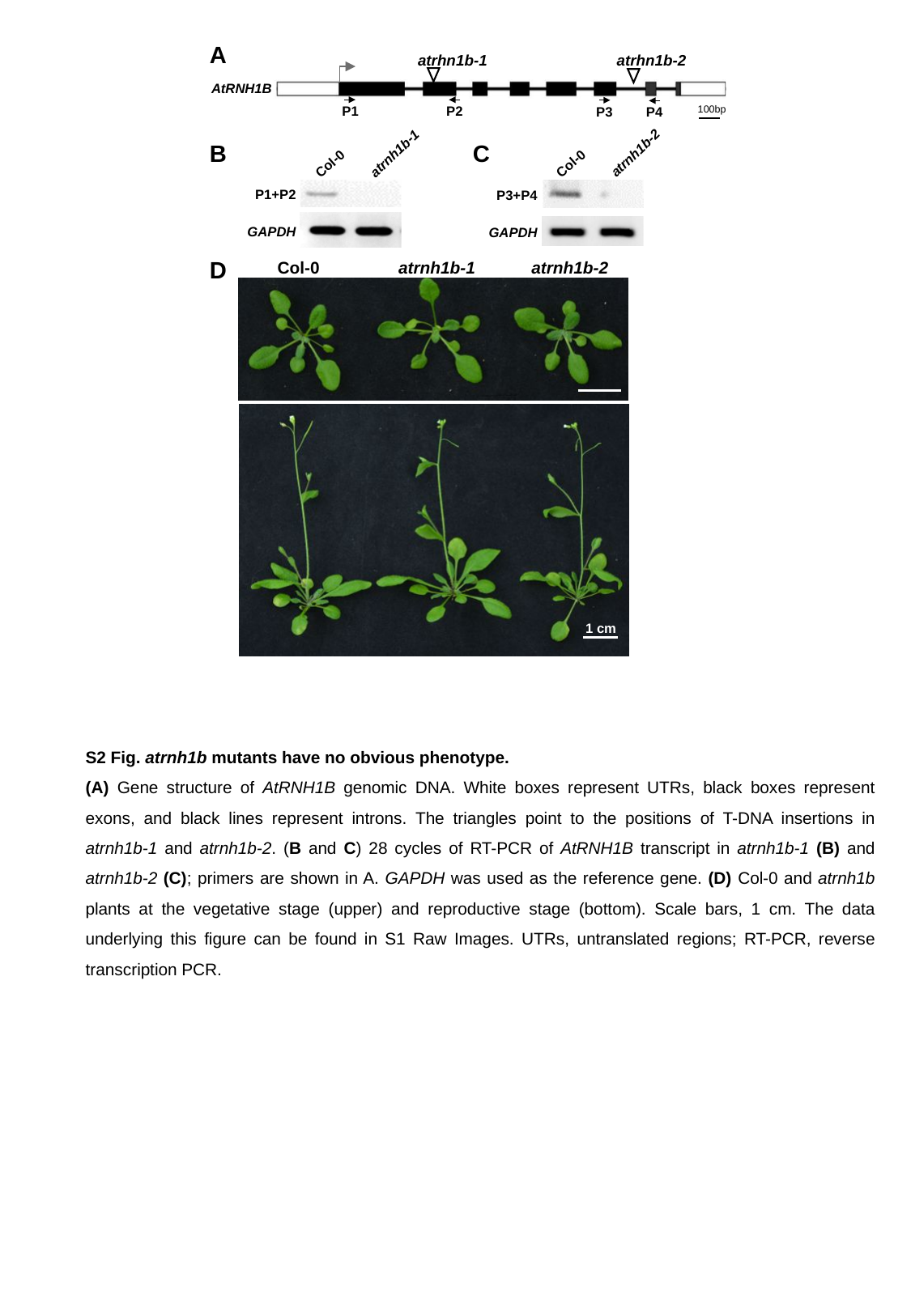

A
atrhn1b-1
atrhn1b-2
AtRNH1B
100bp
P2
P1
P3
P4
B
C
atrnh1b-2
Col-0
P3+P4
GAPDH
atrnh1b-1
Col-0
P1+P2
GAPDH
D
Col-0
atrnh1b-1
atrnh1b-2
1 cm
S2 Fig. atrnh1b mutants have no obvious phenotype.
(A) Gene structure of AtRNH1B genomic DNA. White boxes represent UTRs, black boxes represent exons, and black lines represent introns. The triangles point to the positions of T-DNA insertions in atrnh1b-1 and atrnh1b-2. (B and C) 28 cycles of RT-PCR of AtRNH1B transcript in atrnh1b-1 (B) and atrnh1b-2 (C); primers are shown in A. GAPDH was used as the reference gene. (D) Col-0 and atrnh1b plants at the vegetative stage (upper) and reproductive stage (bottom). Scale bars, 1 cm. The data underlying this figure can be found in S1 Raw Images. UTRs, untranslated regions; RT-PCR, reverse transcription PCR.
