# Supplementary material for: Mitochondrial RNase H1 activity regulates R-loop homeostasis to maintain genome integrity and enable early embryogenesis in Arabidopsis
Source: PLoS Biol. 2021 Aug 3;19(8):e3001357. doi: 10.1371/journal.pbio.3001357 (PMC8330923; doi:10.1371/journal.pbio.3001357)
Supplement: S3 Fig — (A) Representative siliques of atrnh1b-1 and atrnh1c plants, and siliques from reciprocal crosses of Col-0 and atrnh1b 1c+/−. Scale bars, 200 μm. (B) The ratio of atrnh1b 1c+/− to atrnh1b-1 was examined in 50 plates, each containing approximately 150 plants. Seeds were harvested from atrnh1b 1c+/− plants, sown on 1/2 MS medium, and selected based on Basta resistance. Because the atrnh1c mutant is resistant to Basta, the live plants are atrnh1b 1c+/−, and the dead plants are atrnh1b-1. (C) F2 plants of atrnh1b 1c+/− complemented with AtRNH1Bpro:AtRNH1B/AtRNH1BΔMTS-GFP that were sown on 1/2 MS medium and selected based on Basta and hygromycin resistance. The complemented plants are resistant to hygromycin. The green plants are heterozygous atrnh1b 1c+/−, and the yellow plants are homozygous atrnh1b/c. (D) Different phenotypes of embryos in abnormal seeds that failed to transition to the heart stage. Scale bars, 20 μm. (E) Transmission electron microscopy of globular embryos from Col-0 and atrnh1c plants. White arrowheads indicate internal cristae membranes. Scale bars, 500 nm. (F) Confocal microscopy of mitochondria (labeled with MitoTracker) in atrnh1b/c aborted seeds and Col-0 normal seeds. Magenta = MitoTracker. Scale bars, 10 μm. The data underlying this figure can be found in S1 Data. 1/2 MS, half strength Murashige & Skoog (MS); GFP, green fluorescent protein; MTS, mitochondrial targeting signal. (PPTX) [file pbio.3001357.s003.pptx]

## Slide 1
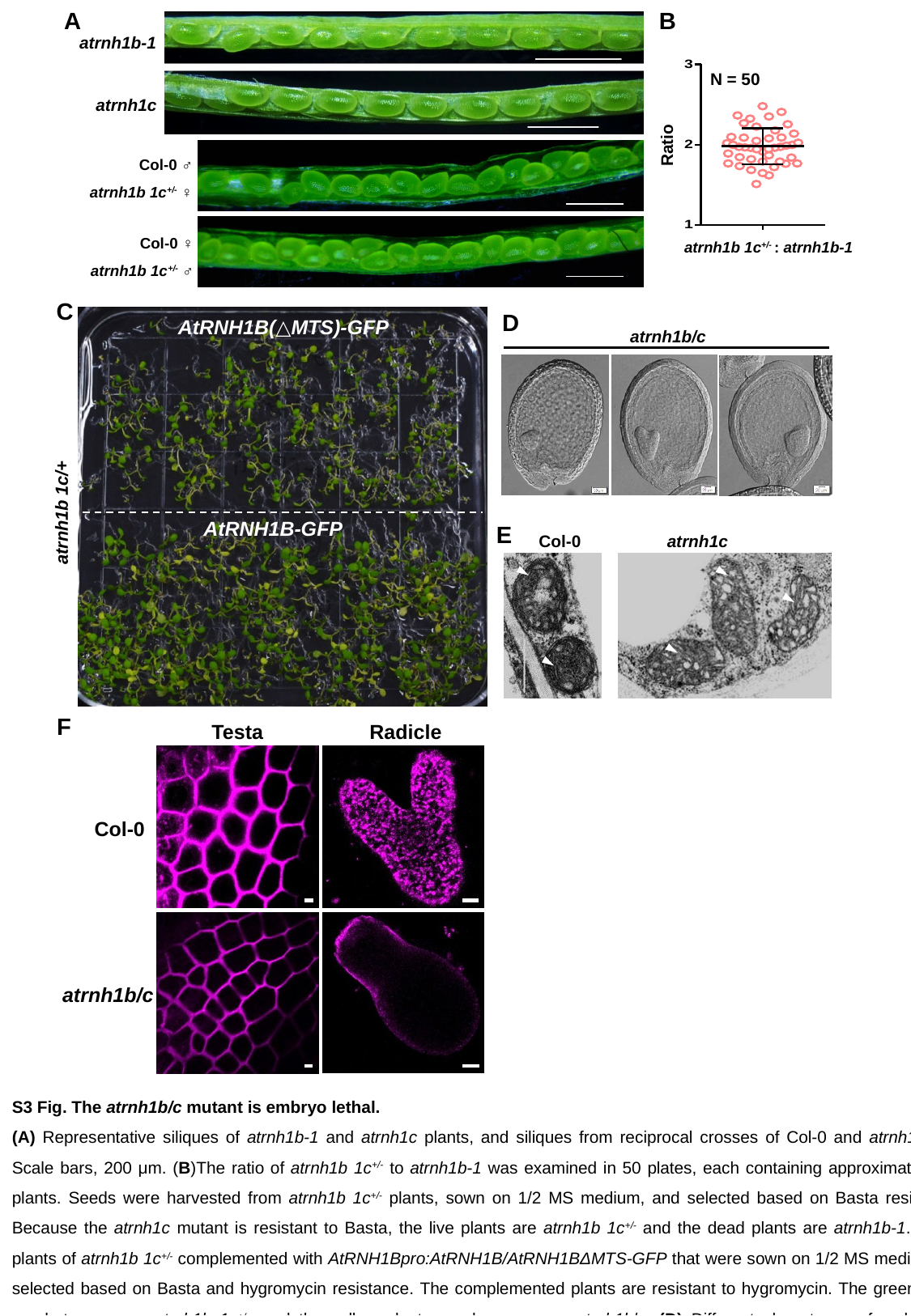

A
B
atrnh1b-1
N = 50
Ratio
atrnh1b 1c+/- : atrnh1b-1
atrnh1c
Col-0 ♂
atrnh1b 1c+/- ♀
Col-0 ♀
atrnh1b 1c+/- ♂
C
D
AtRNH1B(△MTS)-GFP
atrnh1b/c
atrnh1b 1c/+
AtRNH1B-GFP
E
Col-0
atrnh1c
F
Testa
Radicle
Col-0
atrnh1b/c
S3 Fig. The atrnh1b/c mutant is embryo lethal.
(A) Representative siliques of atrnh1b-1 and atrnh1c plants, and siliques from reciprocal crosses of Col-0 and atrnh1b 1c+/-. Scale bars, 200 μm. (B)The ratio of atrnh1b 1c+/- to atrnh1b-1 was examined in 50 plates, each containing approximately 150 plants. Seeds were harvested from atrnh1b 1c+/- plants, sown on 1/2 MS medium, and selected based on Basta resistance. Because the atrnh1c mutant is resistant to Basta, the live plants are atrnh1b 1c+/- and the dead plants are atrnh1b-1. (C) F2 plants of atrnh1b 1c+/- complemented with AtRNH1Bpro:AtRNH1B/AtRNH1BΔMTS-GFP that were sown on 1/2 MS medium and selected based on Basta and hygromycin resistance. The complemented plants are resistant to hygromycin. The green plants are heterozygous atrnh1b 1c+/-, and the yellow plants are homozygous atrnh1b/c. (D) Different phenotypes of embryos in abnormal seeds that failed to transition to the heart stage. Scale bars, 20 μm. (E) Transmission electron microscopy of globular embryos from Col-0 and atrnh1c plants. White arrowheads indicate internal cristae membranes. Scale bars, 500 nm. (F) Confocal microscopy of mitochondria (labeled with MitoTracker) in atrnh1b/c aborted seeds and Col-0 normal seeds. Magenta= MitoTracker. Scale bars, 10 μm. The data underlying this figure can be found in S1 Data. 1/2 MS, half strength Murashige & Skoog (MS); MTS, mitochondrial targeting signal; GFP, green fluorescent protein.
