# Supplementary material for: Mitochondrial RNase H1 activity regulates R-loop homeostasis to maintain genome integrity and enable early embryogenesis in Arabidopsis
Source: PLoS Biol. 2021 Aug 3;19(8):e3001357. doi: 10.1371/journal.pbio.3001357 (PMC8330923; doi:10.1371/journal.pbio.3001357)
Supplement: S4 Fig — (A) Protoplasts from the root tips of AtRNH1C-GFP transgenic plants in atrnh1b-1 background. (B) Sequence alignment of AtRNH1B and AtRNH1C. Red boxes show the 2 deletions in Figs 5 and S5C. Domains are shown above the sequences. (C) Transformed protoplasts from Col-0. (D) GUS staining of different transgenic lines (#1, #2, #3, and #6) of AtRNH1Cpro:AtRNH1C-GUS in the atrnh1b-1 and atrnh1c backgrounds, respectively. CTS, chloroplast targeting signal; GFP, green fluorescent protein; GUS, β-glucuronidase enzyme; HBD, hybrid binding domain; MTS, mitochondrial targeting signal. (PPTX) [file pbio.3001357.s004.pptx]

## Slide 1
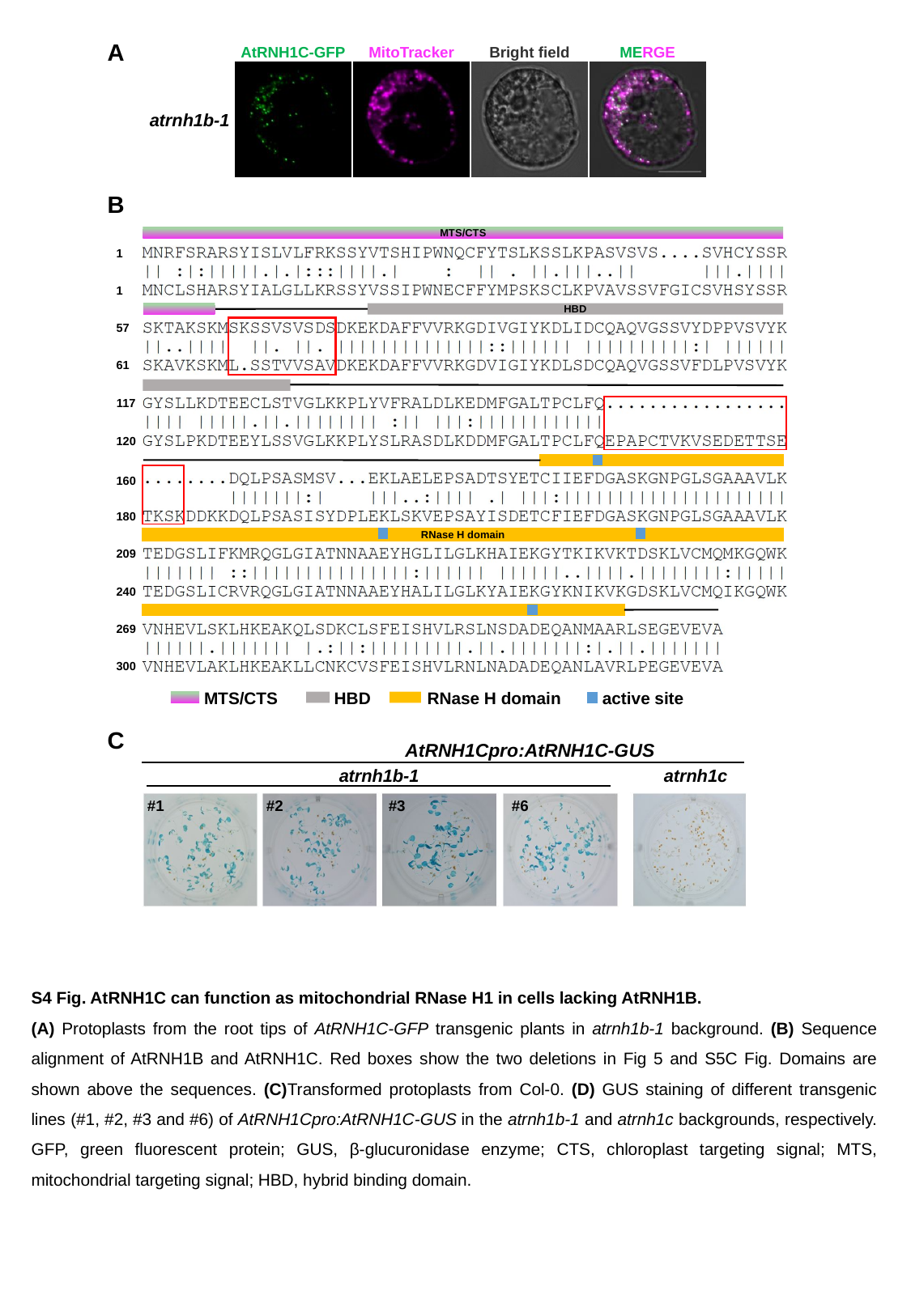

A
AtRNH1C-GFP
MitoTracker
Bright field
MERGE
atrnh1b-1
B
MTS/CTS
HBD
RNase H domain
1
1
57
61
117
120
160
180
209
240
269
300
MTS/CTS
HBD
RNase H domain
active site
C
AtRNH1Cpro:AtRNH1C-GUS
atrnh1c
atrnh1b-1
#1
#2
#3
#6
S4 Fig. AtRNH1C can function as mitochondrial RNase H1 in cells lacking AtRNH1B.
(A) Protoplasts from the root tips of AtRNH1C-GFP transgenic plants in atrnh1b-1 background. (B) Sequence alignment of AtRNH1B and AtRNH1C. Red boxes show the two deletions in Fig 5 and S5C Fig. Domains are shown above the sequences. (C)Transformed protoplasts from Col-0. (D) GUS staining of different transgenic lines (#1, #2, #3 and #6) of AtRNH1Cpro:AtRNH1C-GUS in the atrnh1b-1 and atrnh1c backgrounds, respectively. GFP, green fluorescent protein; GUS, β-glucuronidase enzyme; CTS, chloroplast targeting signal; MTS, mitochondrial targeting signal; HBD, hybrid binding domain.
