# Supplementary material for: Mitochondrial RNase H1 activity regulates R-loop homeostasis to maintain genome integrity and enable early embryogenesis in Arabidopsis
Source: PLoS Biol. 2021 Aug 3;19(8):e3001357. doi: 10.1371/journal.pbio.3001357 (PMC8330923; doi:10.1371/journal.pbio.3001357)
Supplement: S5 Fig — (A) Carboxyl terminus of AtRNH1B cDNA. Blue box indicates the RNAi target sequence. (B) RT-qPCR analysis indicates the reduced expression of AtRNH1B in different AtRNH1BRNAi atrnh1c lines (hereafter, shown as #1–8 for briefness); primers are indicated in A. Data are normalized to atrnh1c and shown as mean values ± SD; circles show the original data of 6 repeats from 2 biological replicates. (C) Seedlings of 3-week-old Col-0, atrnh1b-1, atrnh1c, and AtRNH1BRNAi atrnh1c #1, #2, and #3. (D) Branches of Col-0 and AtRNH1BRNAi atrnh1c plants. (E) Siliques of Col-0 (lower) and AtRNH1BRNAi atrnh1c #1 (upper). Scale bars, 1 cm. (F) Transmission electron microscopy of siliques from Col-0, atrnh1c, and AtRNH1BRNAi atrnh1c plants. Scale bars, 1 μm. (G) Pollen viability in 5-week-old plants by Alexander staining. Scale bars, 10 μm. (H) Siliques from reciprocal crosses of Col-0 and AtRNH1BRNAi atrnh1c #1. Scale bars, 200 μm. The data underlying this figure can be found in S1 Data. RNAi, RNA interference; RT-qPCR, reverse transcription quantitative PCR; SD, standard deviation. (PPTX) [file pbio.3001357.s005.pptx]

## Slide 1
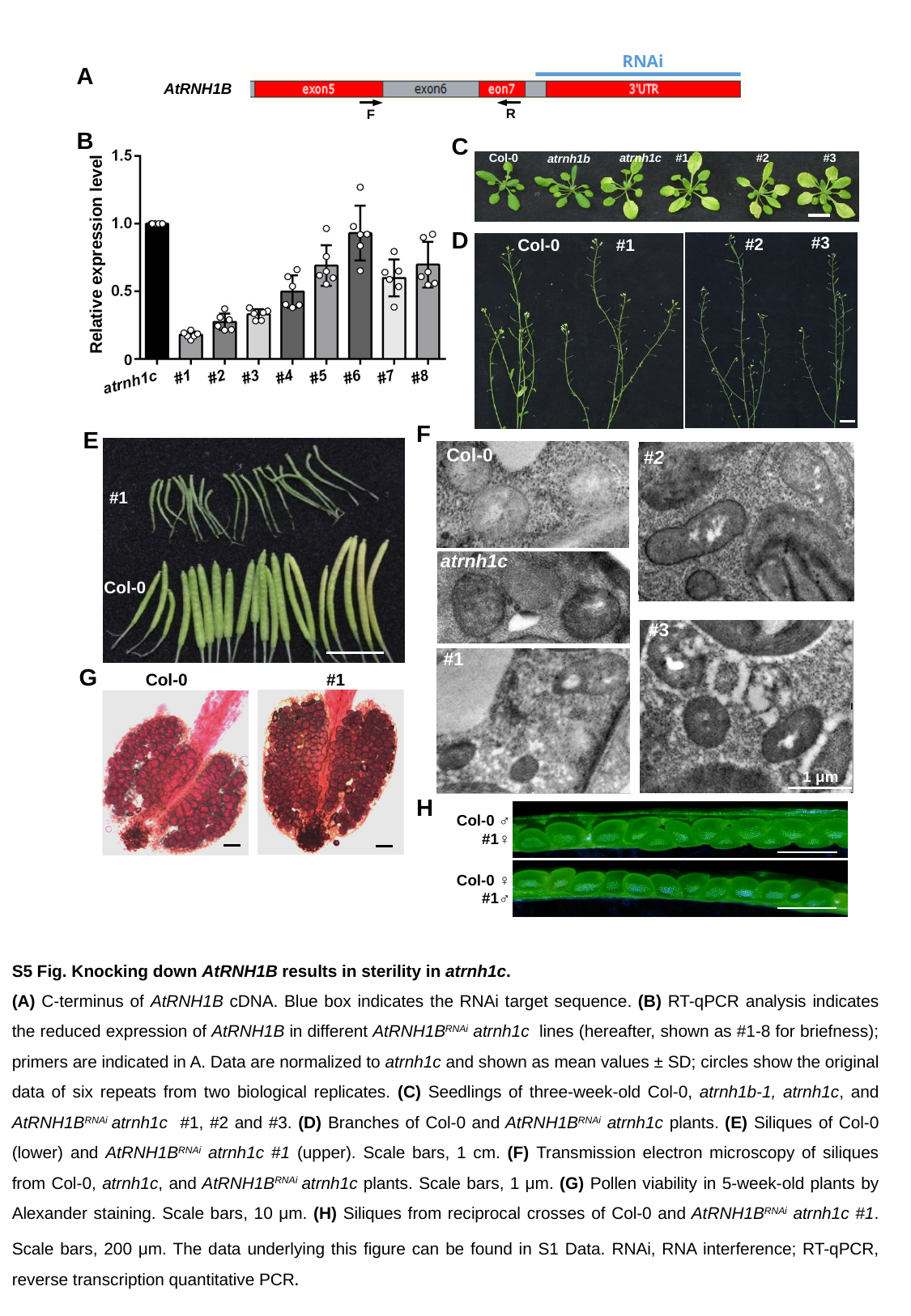

RNAi
A
AtRNH1B
R
F
B
C
Col-0
atrnh1c
#1
#2
#3
atrnh1b
D
#3
#2
Col-0
#1
Relative expression level
0
F
E
Col-0
atrnh1c
1 μm
#3
#2
#1
#1
Col-0
G
Col-0
#1
H
Col-0 ♂
#1♀
Col-0 ♀
#1♂
S5 Fig. Knocking down AtRNH1B results in sterility in atrnh1c.
(A) C-terminus of AtRNH1B cDNA. Blue box indicates the RNAi target sequence. (B) RT-qPCR analysis indicates the reduced expression of AtRNH1B in different AtRNH1BRNAi atrnh1c lines (hereafter, shown as #1-8 for briefness); primers are indicated in A. Data are normalized to atrnh1c and shown as mean values ± SD; circles show the original data of six repeats from two biological replicates. (C) Seedlings of three-week-old Col-0, atrnh1b-1, atrnh1c, and AtRNH1BRNAi atrnh1c #1, #2 and #3. (D) Branches of Col-0 and AtRNH1BRNAi atrnh1c plants. (E) Siliques of Col-0 (lower) and AtRNH1BRNAi atrnh1c #1 (upper). Scale bars, 1 cm. (F) Transmission electron microscopy of siliques from Col-0, atrnh1c, and AtRNH1BRNAi atrnh1c plants. Scale bars, 1 μm. (G) Pollen viability in 5-week-old plants by Alexander staining. Scale bars, 10 μm. (H) Siliques from reciprocal crosses of Col-0 and AtRNH1BRNAi atrnh1c #1. Scale bars, 200 μm. The data underlying this figure can be found in S1 Data. RNAi, RNA interference; RT-qPCR, reverse transcription quantitative PCR.
