# Supplementary material for: Mitochondrial RNase H1 activity regulates R-loop homeostasis to maintain genome integrity and enable early embryogenesis in Arabidopsis
Source: PLoS Biol. 2021 Aug 3;19(8):e3001357. doi: 10.1371/journal.pbio.3001357 (PMC8330923; doi:10.1371/journal.pbio.3001357)
Supplement: S6 Fig — (A) Schematic map of Arabidopsis mtDNA. Six repeat pairs are indicated in the map. (B) Relative quantification of copy numbers of mtDNA (18S, COX2) gene sequences in different tissues of Col-0. Nuclear genes UBC and ACT2 were used as reference genes. (C) Relative quantification of the crossover products (as depicted in Fig 5B) of L and EE in different tissues of Col-0. Mitochondrial genes 18S and COX2 were used as reference genes. Data are normalized to rosette leaves and shown as mean values ± SD; circles show the original data of 6 repeats containing 2 biological replicates. One-way ANOVA compared to rosette leaves for each locus. *, p < 0.05; **, p < 0.01; ***, p < 0.001; ****, p < 0.0001. others not shown are not significant. The data underlying this figure can be found in S1 Data. ACT2, ACTIN 2; ANOVA, analysis of variance; COX2, CYTOCHROME OXIDASE 2; HR, homologous recombination; mtDNA, mitochondrial DNA; SD, standard deviation; UBC, UBIQUITIN-CONJUGATING ENZYME. (PPTX) [file pbio.3001357.s006.pptx]

## Slide 1
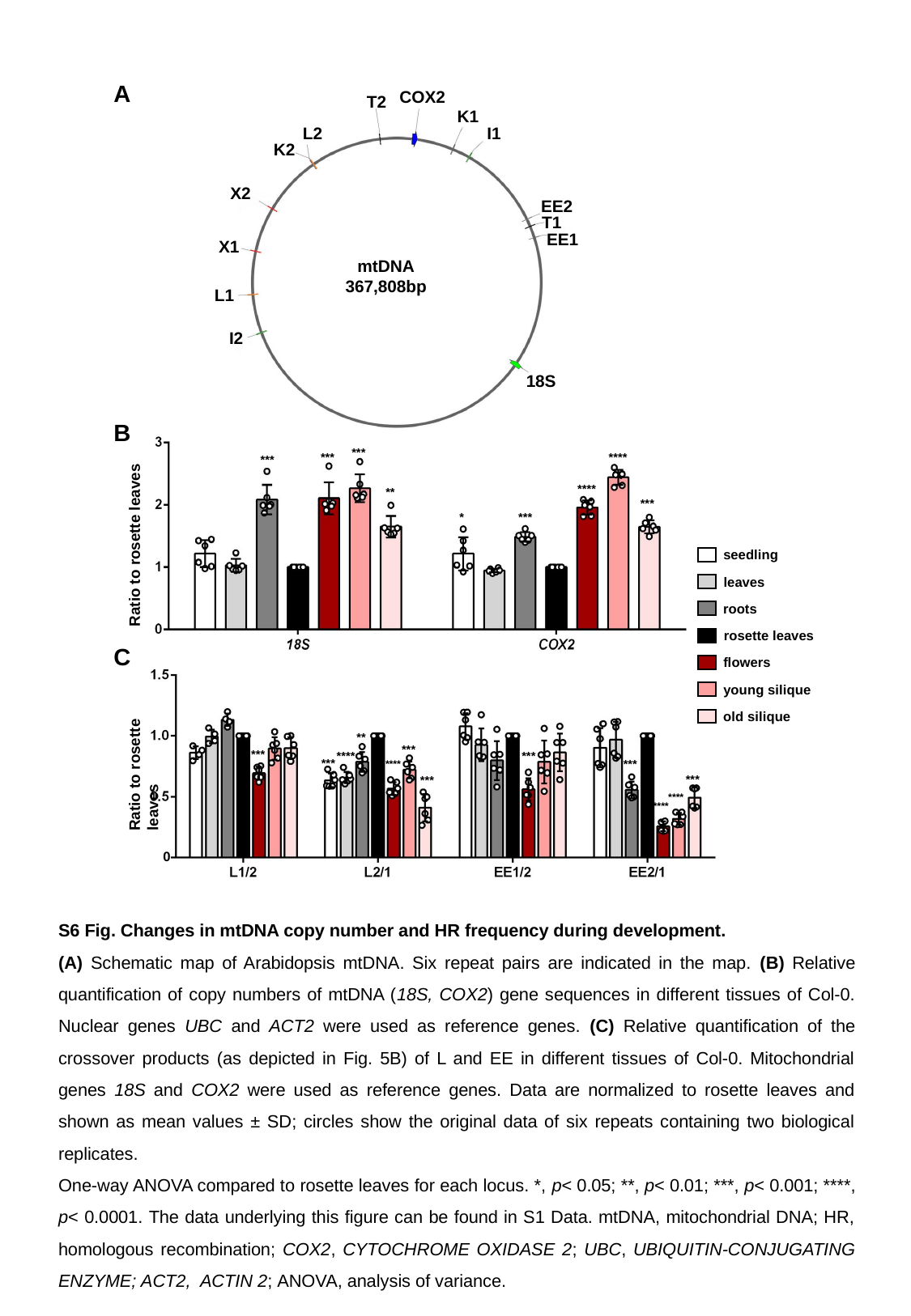

A
COX2
T2
K1
L2
I1
K2
X2
EE2
T1
EE1
X1
mtDNA
367,808bp
L1
I2
18S
B
***
***
****
***
Ratio to rosette leaves
****
**
***
***
*
seedling
leaves
roots
rosette leaves
C
flowers
Ratio to rosette leaves
young silique
old silique
**
***
***
****
***
***
***
****
***
***
****
****
0
S6 Fig. Changes in mtDNA copy number and HR frequency during development.
(A) Schematic map of Arabidopsis mtDNA. Six repeat pairs are indicated in the map. (B) Relative quantification of copy numbers of mtDNA (18S, COX2) gene sequences in different tissues of Col-0. Nuclear genes UBC and ACT2 were used as reference genes. (C) Relative quantification of the crossover products (as depicted in Fig. 5B) of L and EE in different tissues of Col-0. Mitochondrial genes 18S and COX2 were used as reference genes. Data are normalized to rosette leaves and shown as mean values ± SD; circles show the original data of six repeats containing two biological replicates.
One-way ANOVA compared to rosette leaves for each locus. *, p< 0.05; **, p< 0.01; ***, p< 0.001; ****, p< 0.0001. The data underlying this figure can be found in S1 Data. mtDNA, mitochondrial DNA; HR, homologous recombination; COX2, CYTOCHROME OXIDASE 2; UBC, UBIQUITIN-CONJUGATING ENZYME; ACT2,  ACTIN 2; ANOVA, analysis of variance.
