# Supplementary material for: Mitochondrial RNase H1 activity regulates R-loop homeostasis to maintain genome integrity and enable early embryogenesis in Arabidopsis
Source: PLoS Biol. 2021 Aug 3;19(8):e3001357. doi: 10.1371/journal.pbio.3001357 (PMC8330923; doi:10.1371/journal.pbio.3001357)
Supplement: S7 Fig — (A, B) Gene structures of OSB1 (A) and RECA3 (B) genomic DNA. gRNAs for CRISPR knockout are indicated as red lines. (C) DNA gel shows the results of CRISPR knockout. Primers for PCR are shown as osb1-F/R and recA3-F/R in (A). The size of PCR products with osb1-F+R is 342 bp in Col-0 and 163 bp in osb1 and osb1 AtRNH1BRNAi atrnh1c (shown as osb1 RNAi #1 for briefness). The size of PCR products with recA3-F+R is 532 bp in Col-0 and 240 bp in recA3. (D) Sequence chromatograms showing the CRISPR/Cas-9–mediated deletion of OSB1. (E) Sequence chromatograms showing the CRISPR/Cas-9–mediated deletion of RECA3. The data underlying this figure can be found in S1 Raw Images. Cas-9, CRISPR associated protein-9; CRISPR, clustered regularly interspaced short palindromic repeats; F, forward; gRNA, guide RNA; OSB1, ORGANELLAR SINGLE-STRANDED DNA BINDING PROTEIN1; R, reverse. (PPTX) [file pbio.3001357.s007.pptx]

## Slide 1
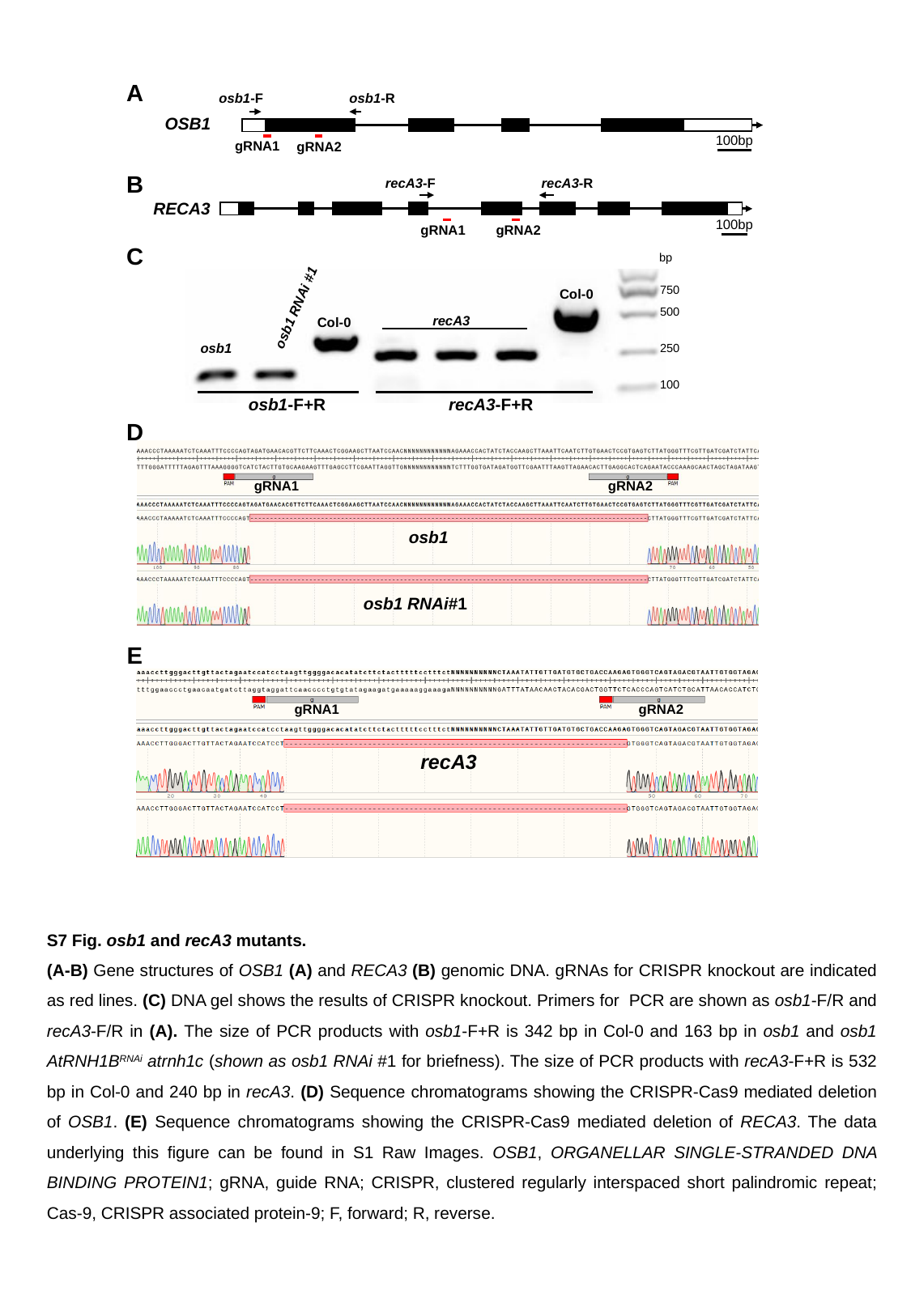

A
osb1-F
osb1-R
OSB1
100bp
gRNA1
gRNA2
B
recA3-R
recA3-F
RECA3
100bp
gRNA1
gRNA2
C
bp
750
Col-0
osb1 RNAi #1
500
recA3
Col-0
osb1
250
100
osb1-F+R
recA3-F+R
D
osb1
osb1 RNAi#1
gRNA1
gRNA2
E
gRNA1
gRNA2
recA3
S7 Fig. osb1 and recA3 mutants.
(A-B) Gene structures of OSB1 (A) and RECA3 (B) genomic DNA. gRNAs for CRISPR knockout are indicated as red lines. (C) DNA gel shows the results of CRISPR knockout. Primers for PCR are shown as osb1-F/R and recA3-F/R in (A). The size of PCR products with osb1-F+R is 342 bp in Col-0 and 163 bp in osb1 and osb1 AtRNH1BRNAi atrnh1c (shown as osb1 RNAi #1 for briefness). The size of PCR products with recA3-F+R is 532 bp in Col-0 and 240 bp in recA3. (D) Sequence chromatograms showing the CRISPR-Cas9 mediated deletion of OSB1. (E) Sequence chromatograms showing the CRISPR-Cas9 mediated deletion of RECA3. The data underlying this figure can be found in S1 Raw Images. OSB1, ORGANELLAR SINGLE-STRANDED DNA BINDING PROTEIN1; gRNA, guide RNA; CRISPR, clustered regularly interspaced short palindromic repeat; Cas-9, CRISPR associated protein-9; F, forward; R, reverse.
