# Supplementary material for: Mitochondrial RNase H1 activity regulates R-loop homeostasis to maintain genome integrity and enable early embryogenesis in Arabidopsis
Source: PLoS Biol. 2021 Aug 3;19(8):e3001357. doi: 10.1371/journal.pbio.3001357 (PMC8330923; doi:10.1371/journal.pbio.3001357)
Supplement: S1 Movie — LiTone LBS Light-sheet microscopy of root of AtRNH1Bpro:AtRNH1B-GFP atrnh1b transgenic plants. Scale bars, 10 μm. Green = GFP and magenta = MitoTracker. (PPTX) [file pbio.3001357.s008.pptx]

## Slide 1
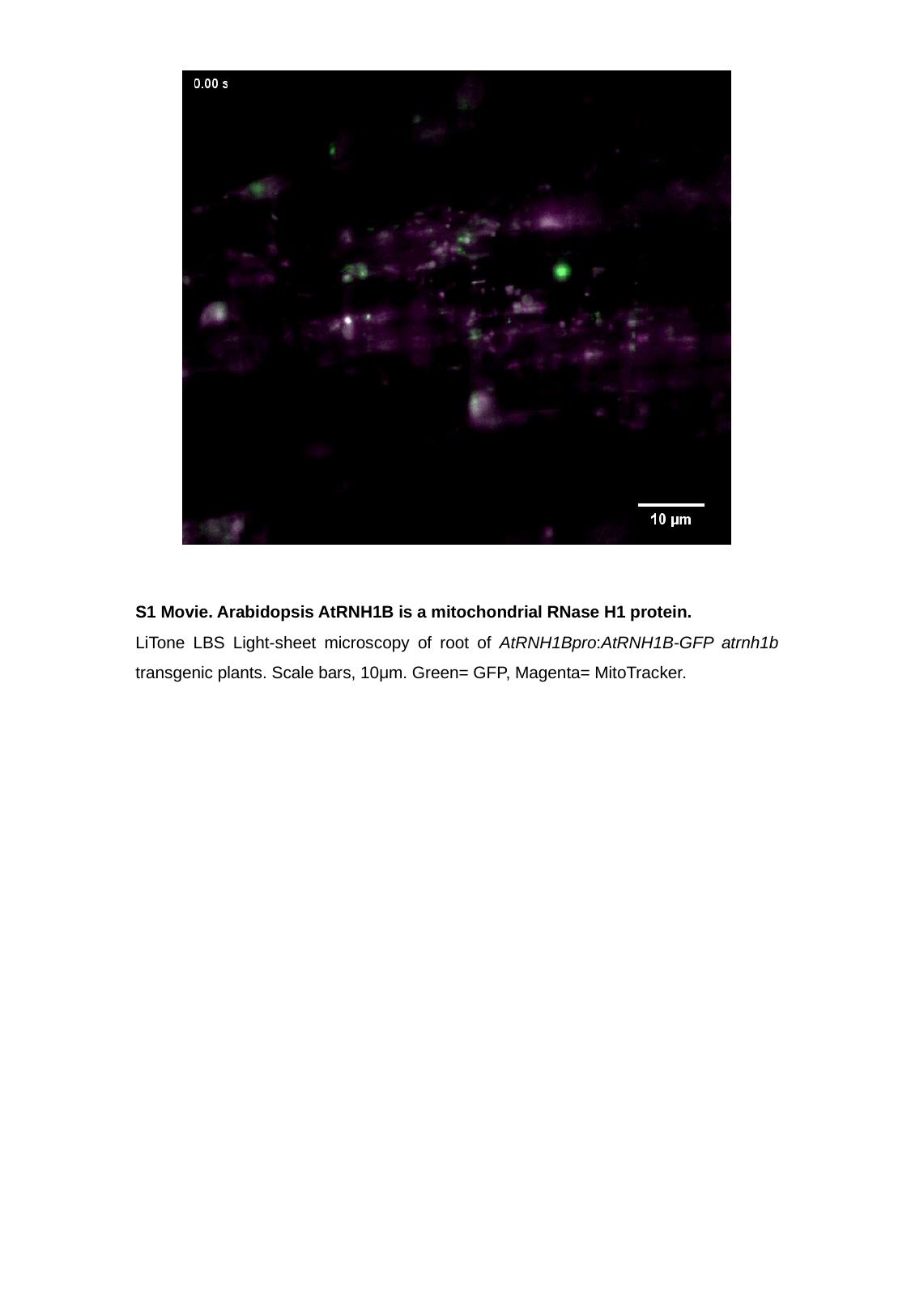

S1 Movie. Arabidopsis AtRNH1B is a mitochondrial RNase H1 protein.
LiTone LBS Light-sheet microscopy of root of AtRNH1Bpro:AtRNH1B-GFP atrnh1b transgenic plants. Scale bars, 10μm. Green= GFP, Magenta= MitoTracker.
