# Supplementary material for: Mitochondrial RNase H1 activity regulates R-loop homeostasis to maintain genome integrity and enable early embryogenesis in Arabidopsis
Source: PLoS Biol. 2021 Aug 3;19(8):e3001357. doi: 10.1371/journal.pbio.3001357 (PMC8330923; doi:10.1371/journal.pbio.3001357)
Supplement: S2 Movie — OLYMPUS DP80 microscope visualizes the GUS signals from the top surface to the bottom of the leaf carrying the AtRNH1Bpro:AtRNH1B-GUS in atrnh1b mutant (complementation lines). (PPTX) [file pbio.3001357.s009.pptx]

## Slide 1
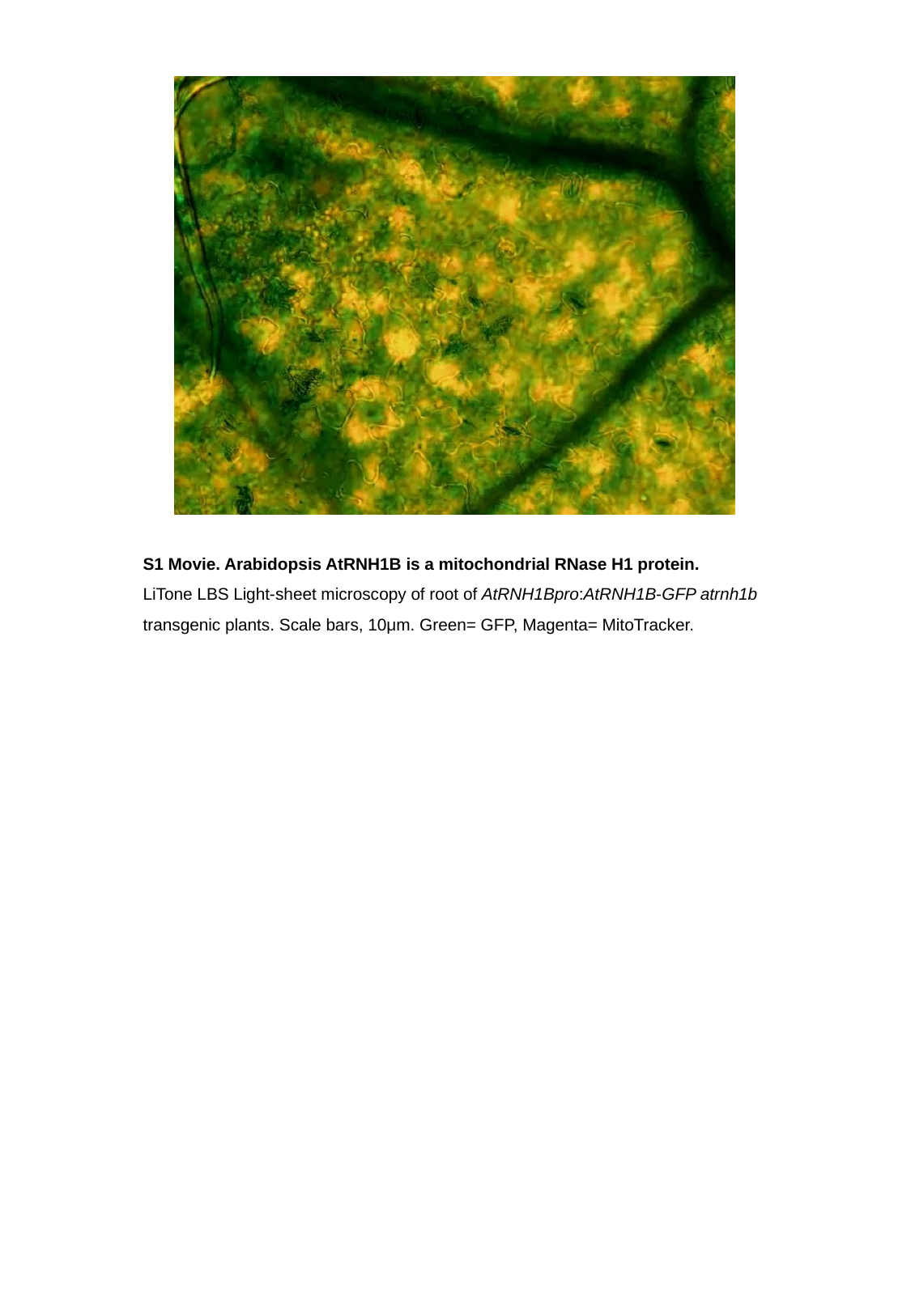

S1 Movie. Arabidopsis AtRNH1B is a mitochondrial RNase H1 protein.
LiTone LBS Light-sheet microscopy of root of AtRNH1Bpro:AtRNH1B-GFP atrnh1b transgenic plants. Scale bars, 10μm. Green= GFP, Magenta= MitoTracker.
