# Supplementary material for: Mitochondrial RNase H1 activity regulates R-loop homeostasis to maintain genome integrity and enable early embryogenesis in Arabidopsis
Source: PLoS Biol. 2021 Aug 3;19(8):e3001357. doi: 10.1371/journal.pbio.3001357 (PMC8330923; doi:10.1371/journal.pbio.3001357)
Supplement: S1 Raw Images — (PPTX) [file pbio.3001357.s012.pptx]

## Slide 1
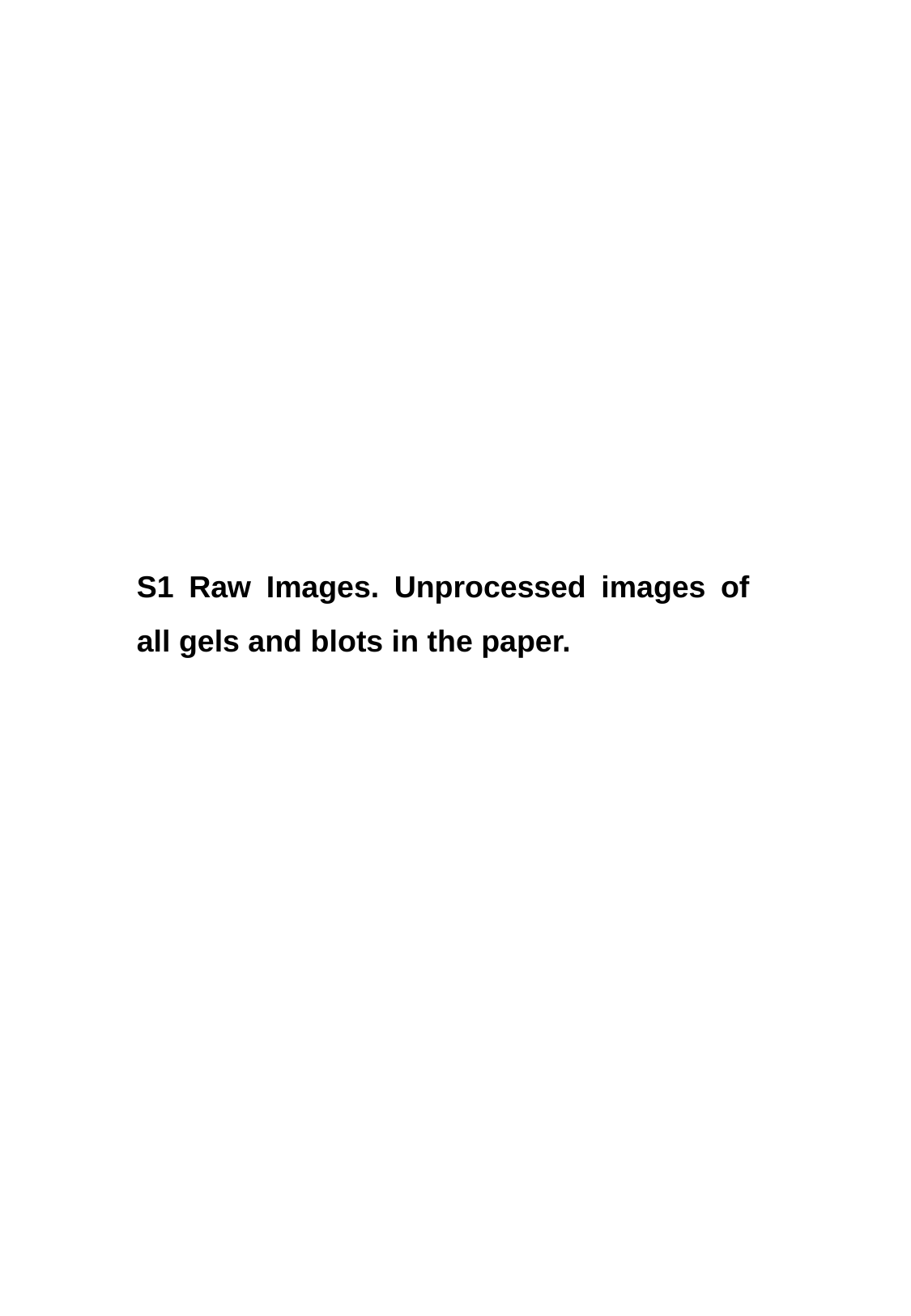

S1 Raw Images. Unprocessed images of all gels and blots in the paper.

## Slide 2
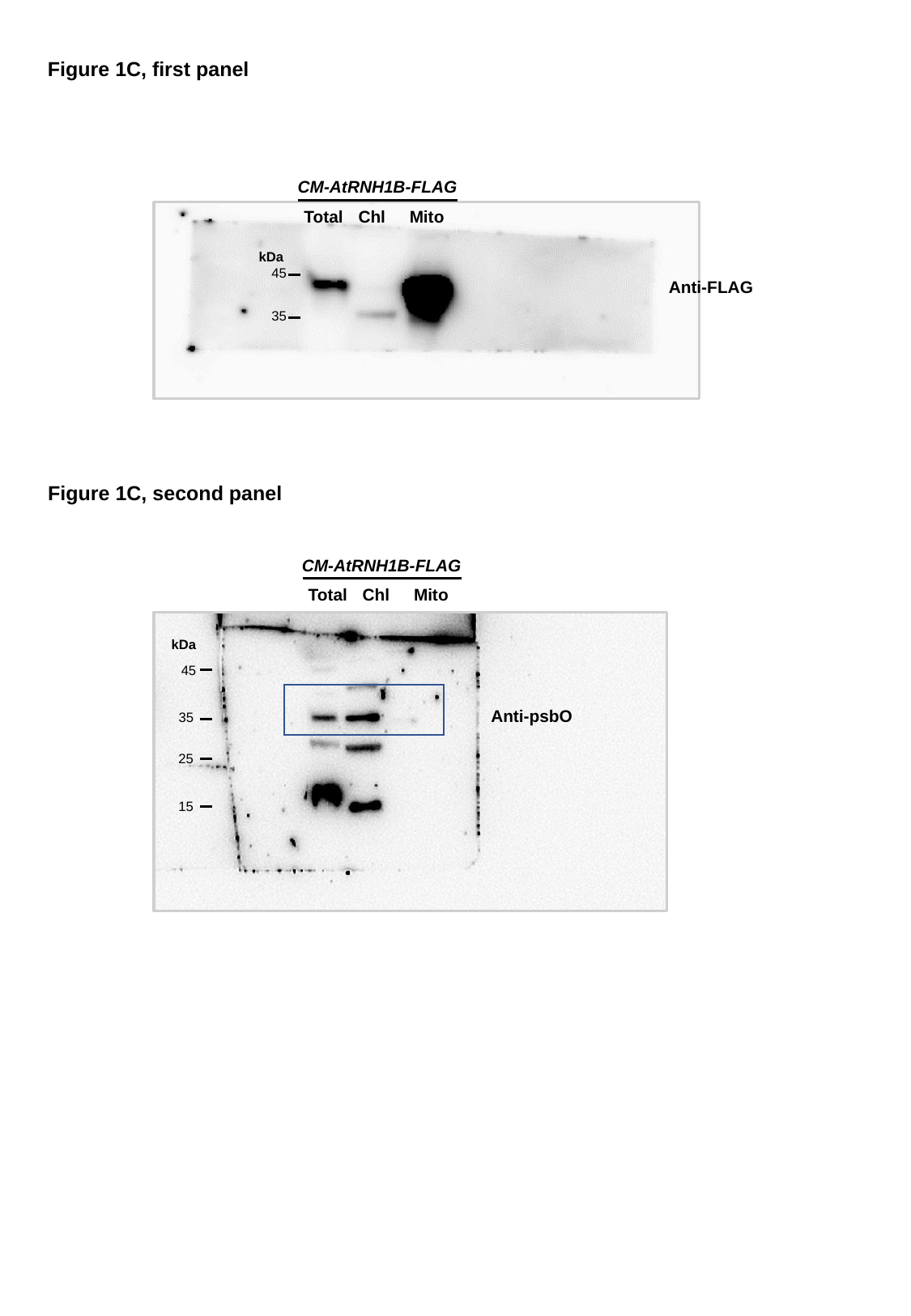

Figure 1C, first panel
CM-AtRNH1B-FLAG
Total
Chl
Mito
kDa
45
Anti-FLAG
35
Figure 1C, second panel
CM-AtRNH1B-FLAG
Total
Chl
Mito
kDa
45
Anti-psbO
35
25
15

## Slide 3
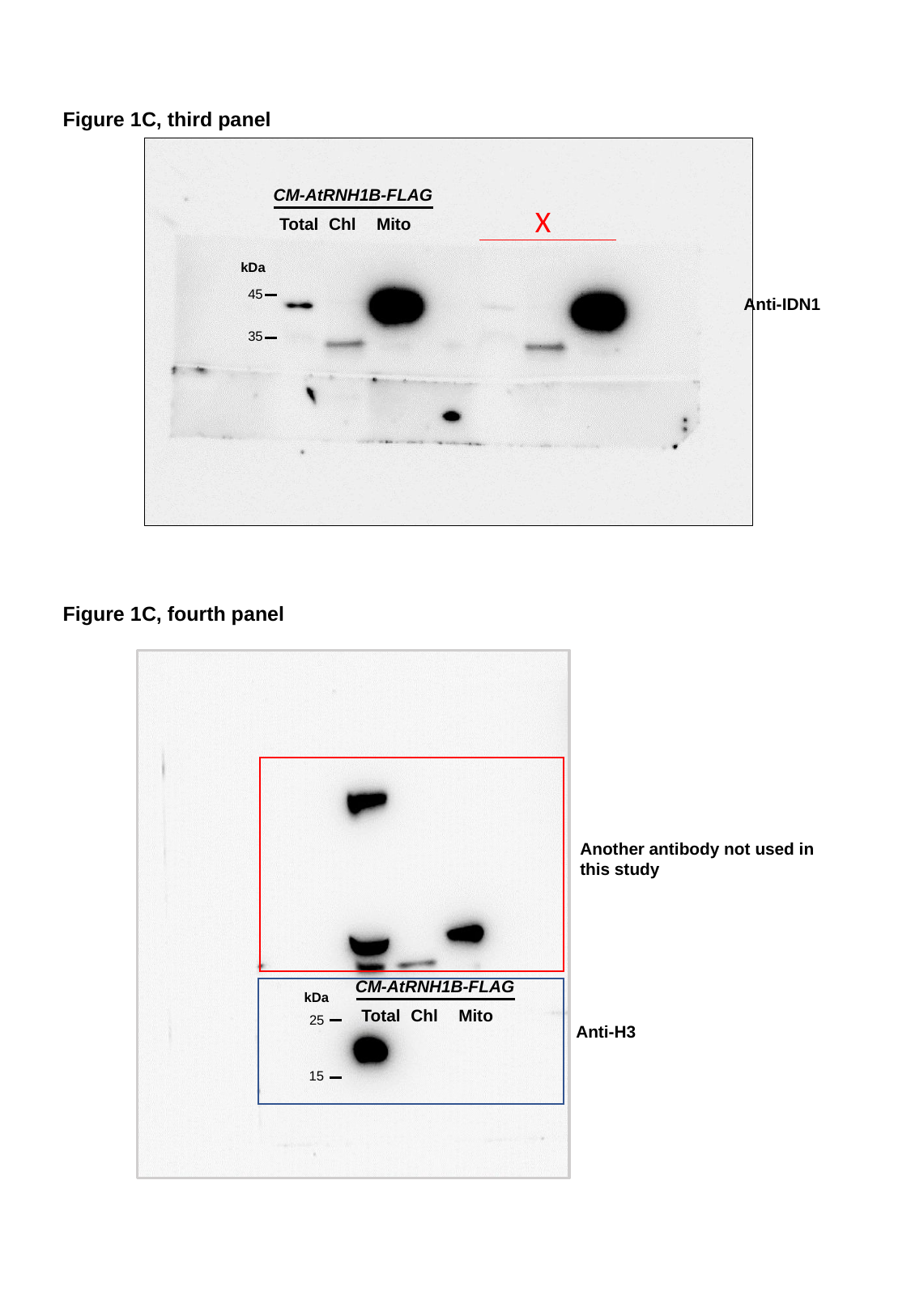

Figure 1C, third panel
CM-AtRNH1B-FLAG
X
Total
Chl
Mito
kDa
45
Anti-IDN1
35
Figure 1C, fourth panel
Another antibody not used in this study
CM-AtRNH1B-FLAG
kDa
Total
Chl
Mito
25
Anti-H3
15

## Slide 4
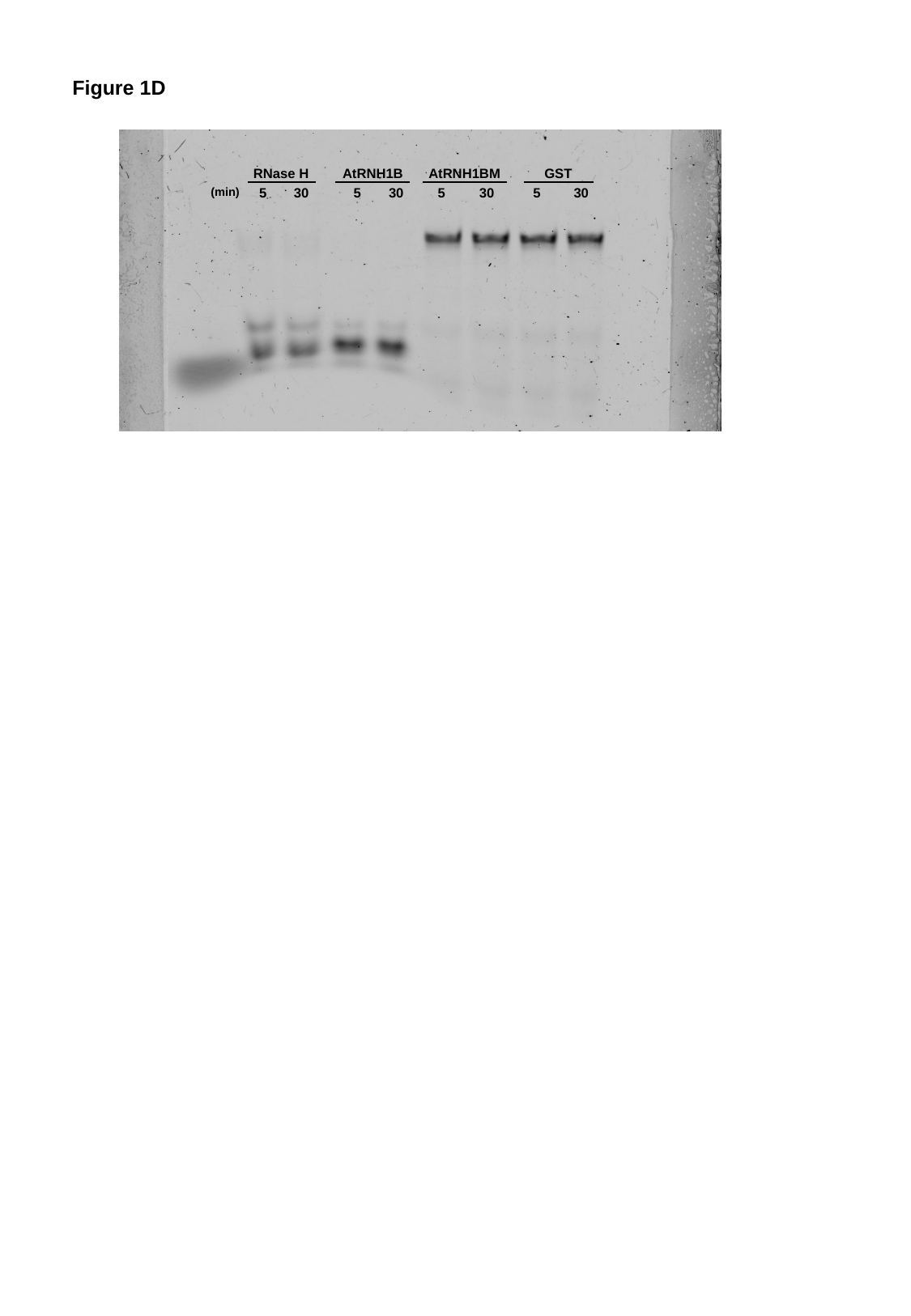

Figure 1D
RNase H
AtRNH1B
AtRNH1BM
GST
5
30
5
30
5
30
5
30
(min)

## Slide 5
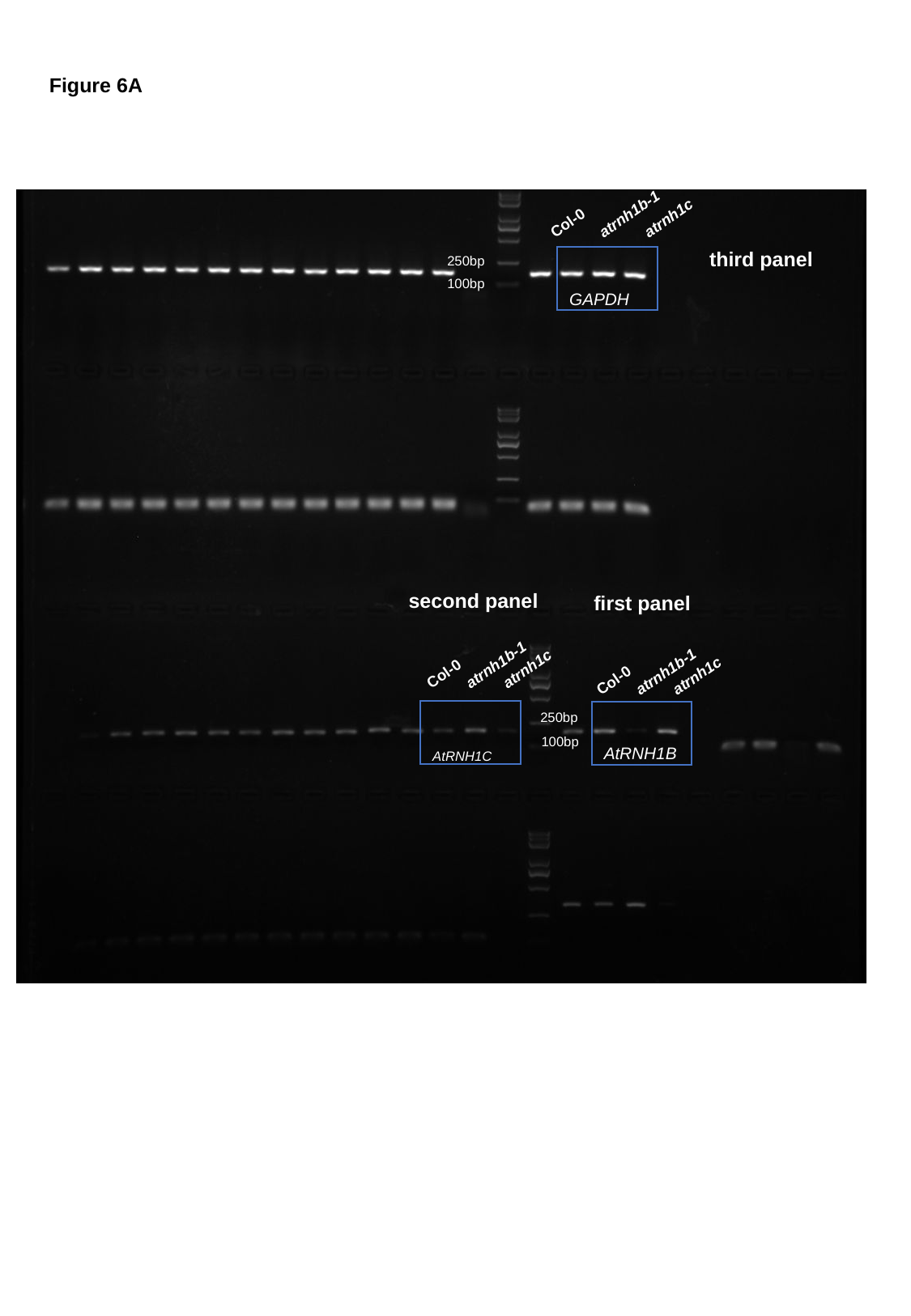

Figure 6A
atrnh1b-1
atrnh1c
Col-0
third panel
250bp
100bp
GAPDH
second panel
first panel
atrnh1b-1
atrnh1c
atrnh1b-1
Col-0
atrnh1c
Col-0
250bp
100bp
AtRNH1B
AtRNH1C

## Slide 6
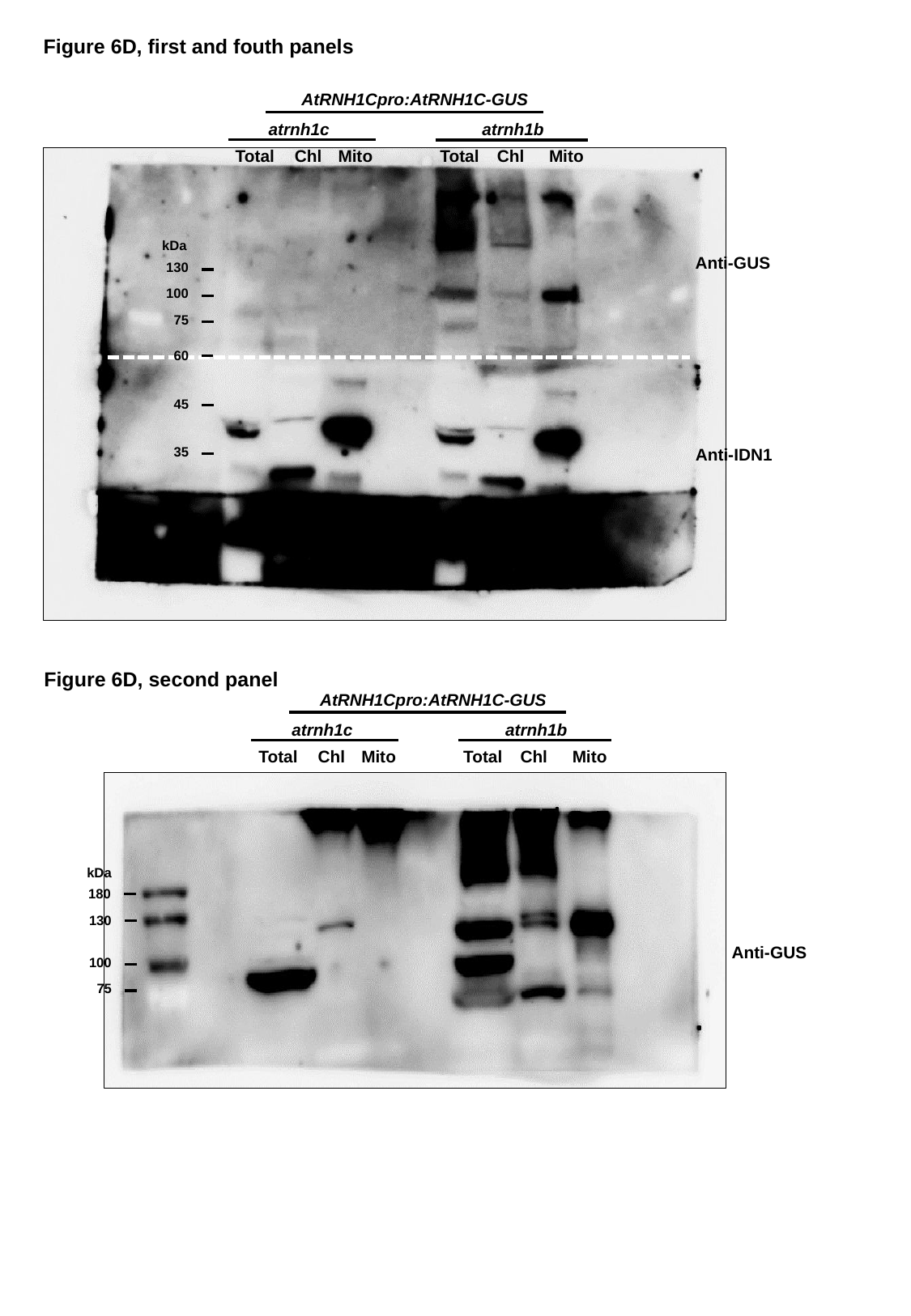

Figure 6D, first and fouth panels
AtRNH1Cpro:AtRNH1C-GUS
atrnh1c
atrnh1b
Total
Chl
Mito
Total
Chl
Mito
kDa
Anti-GUS
130
100
75
60
45
35
Anti-IDN1
Figure 6D, second panel
AtRNH1Cpro:AtRNH1C-GUS
atrnh1c
atrnh1b
Total
Chl
Mito
Total
Chl
Mito
kDa
180
130
Anti-GUS
100
75

## Slide 7
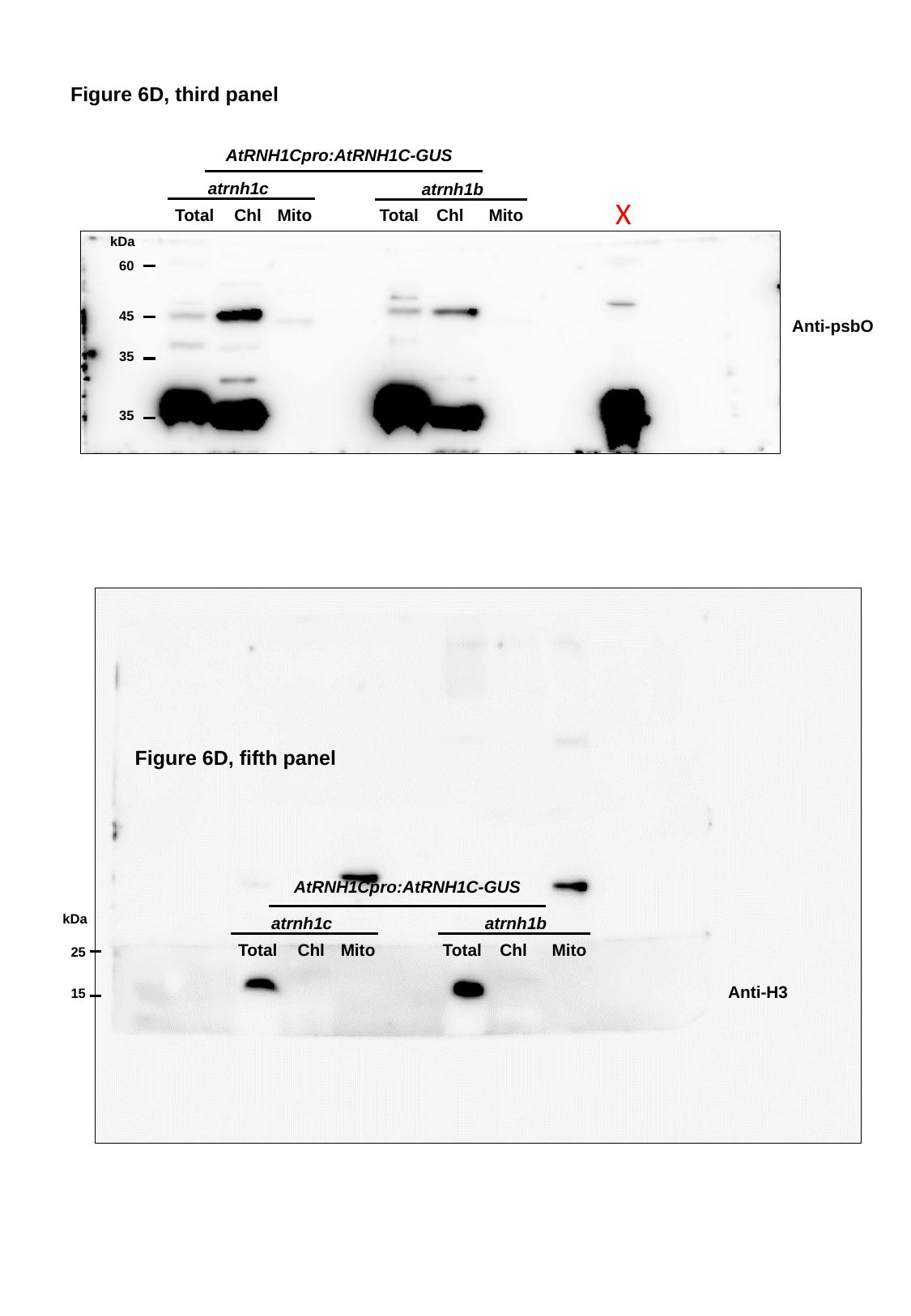

Figure 6D, third panel
AtRNH1Cpro:AtRNH1C-GUS
atrnh1c
atrnh1b
X
Total
Chl
Mito
Total
Chl
Mito
kDa
60
45
Anti-psbO
35
35
Figure 6D, fifth panel
AtRNH1Cpro:AtRNH1C-GUS
kDa
atrnh1c
atrnh1b
Total
Chl
Mito
Total
Chl
Mito
25
Anti-H3
15

## Slide 8
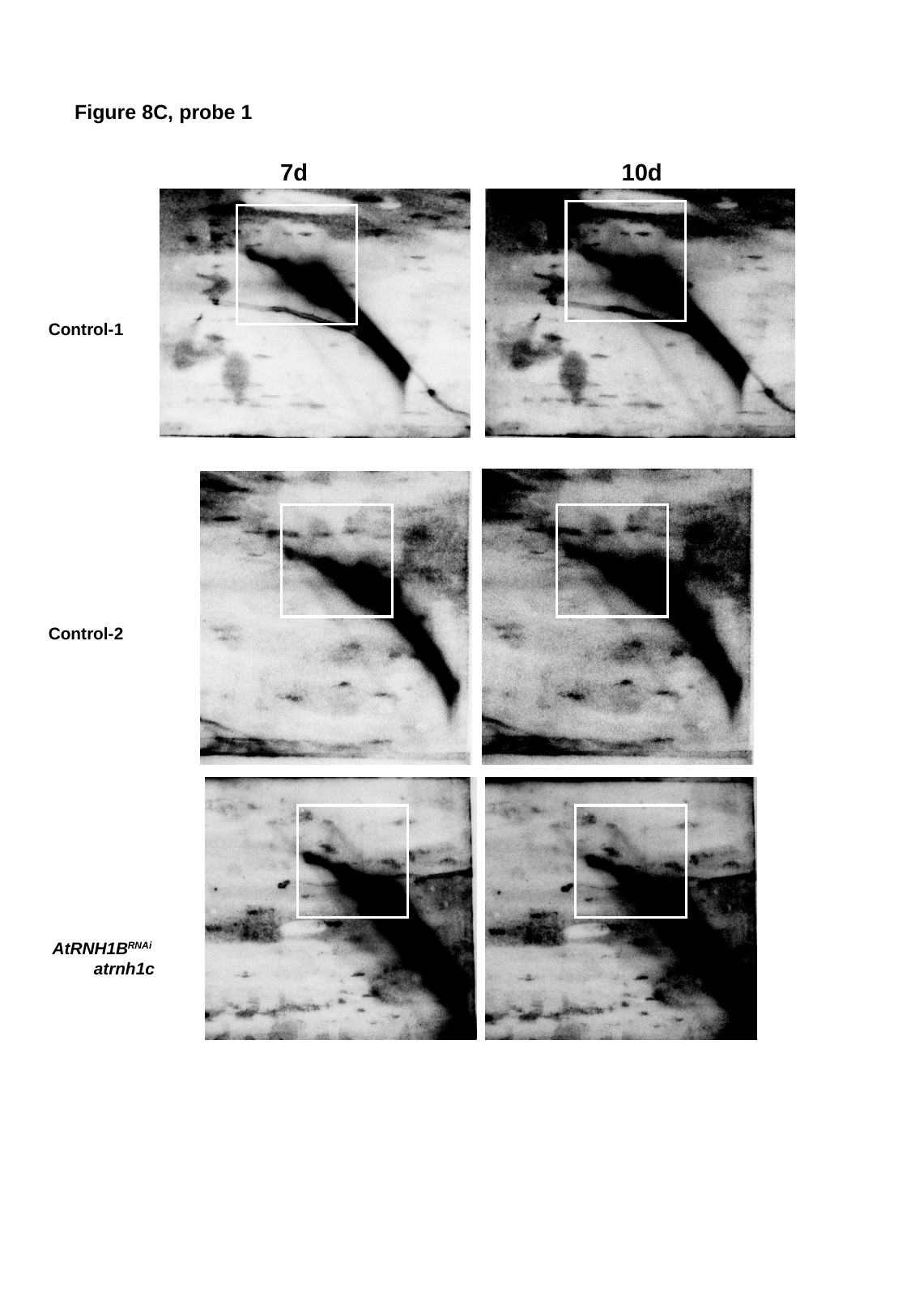

Figure 8C, probe 1
7d
10d
Control-1
Control-2
AtRNH1BRNAi
atrnh1c

## Slide 9
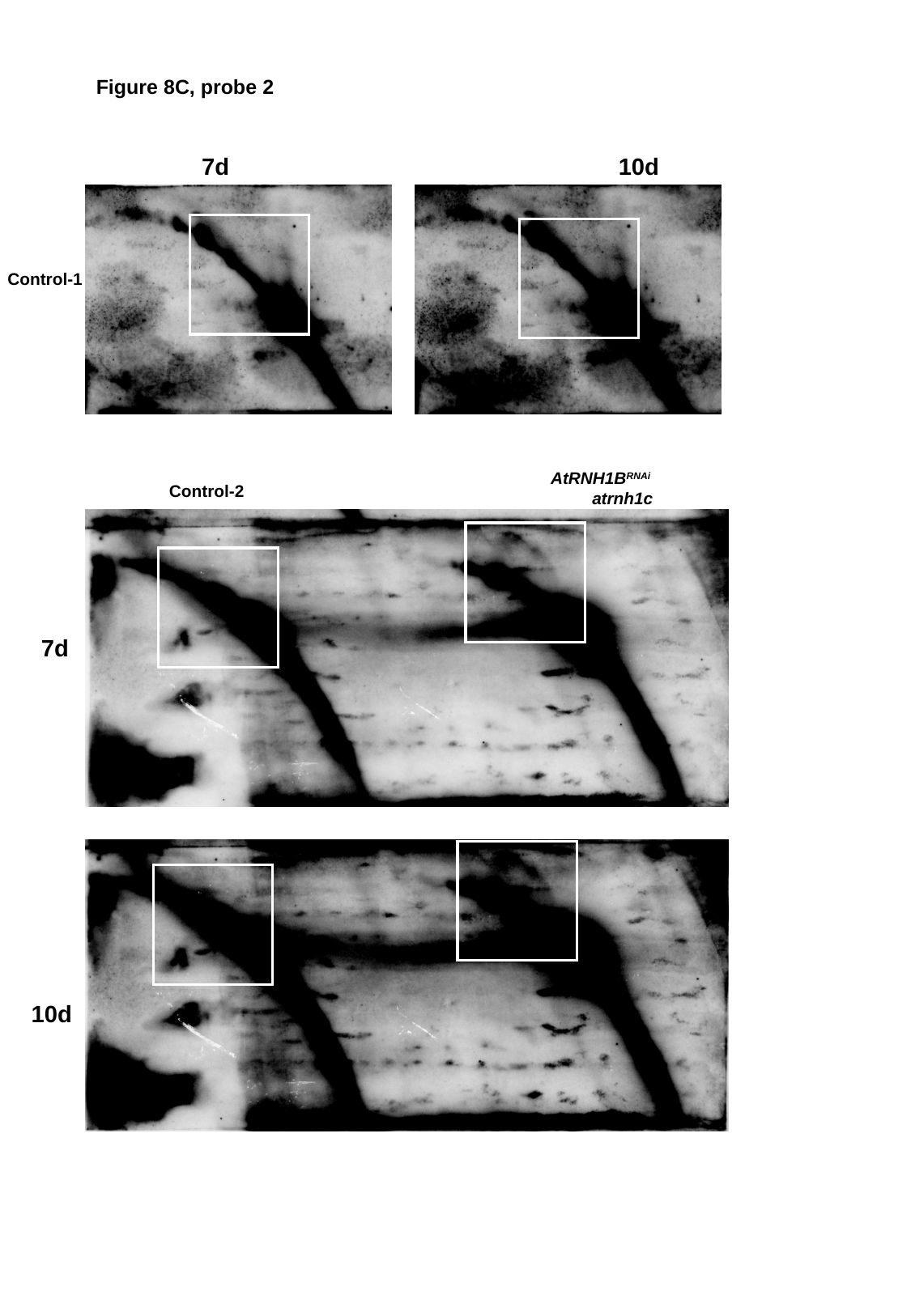

Figure 8C, probe 2
10d
7d
Control-1
AtRNH1BRNAi
atrnh1c
Control-2
7d
10d

## Slide 10
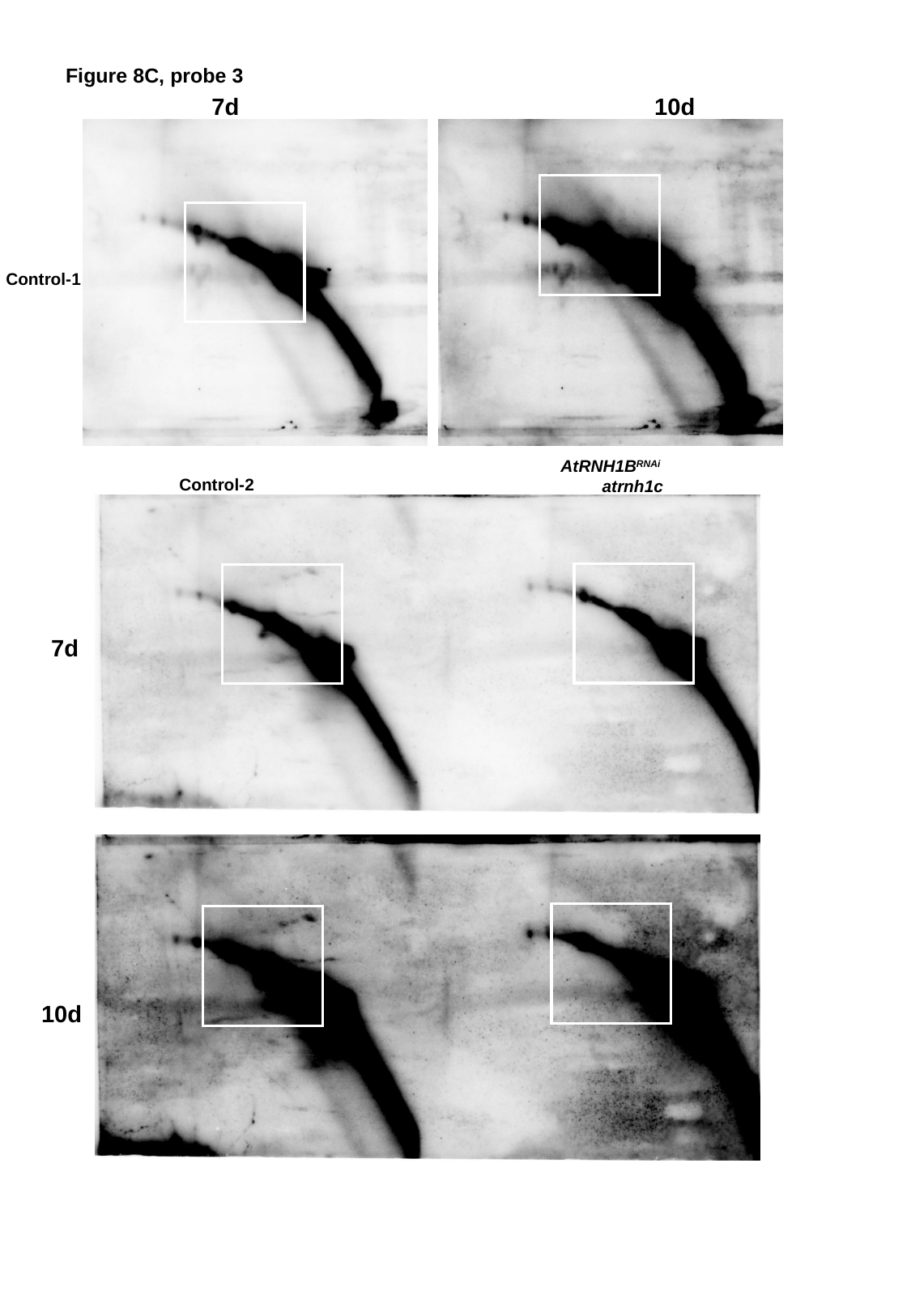

Figure 8C, probe 3
10d
7d
Control-1
AtRNH1BRNAi
atrnh1c
Control-2
7d
10d

## Slide 11
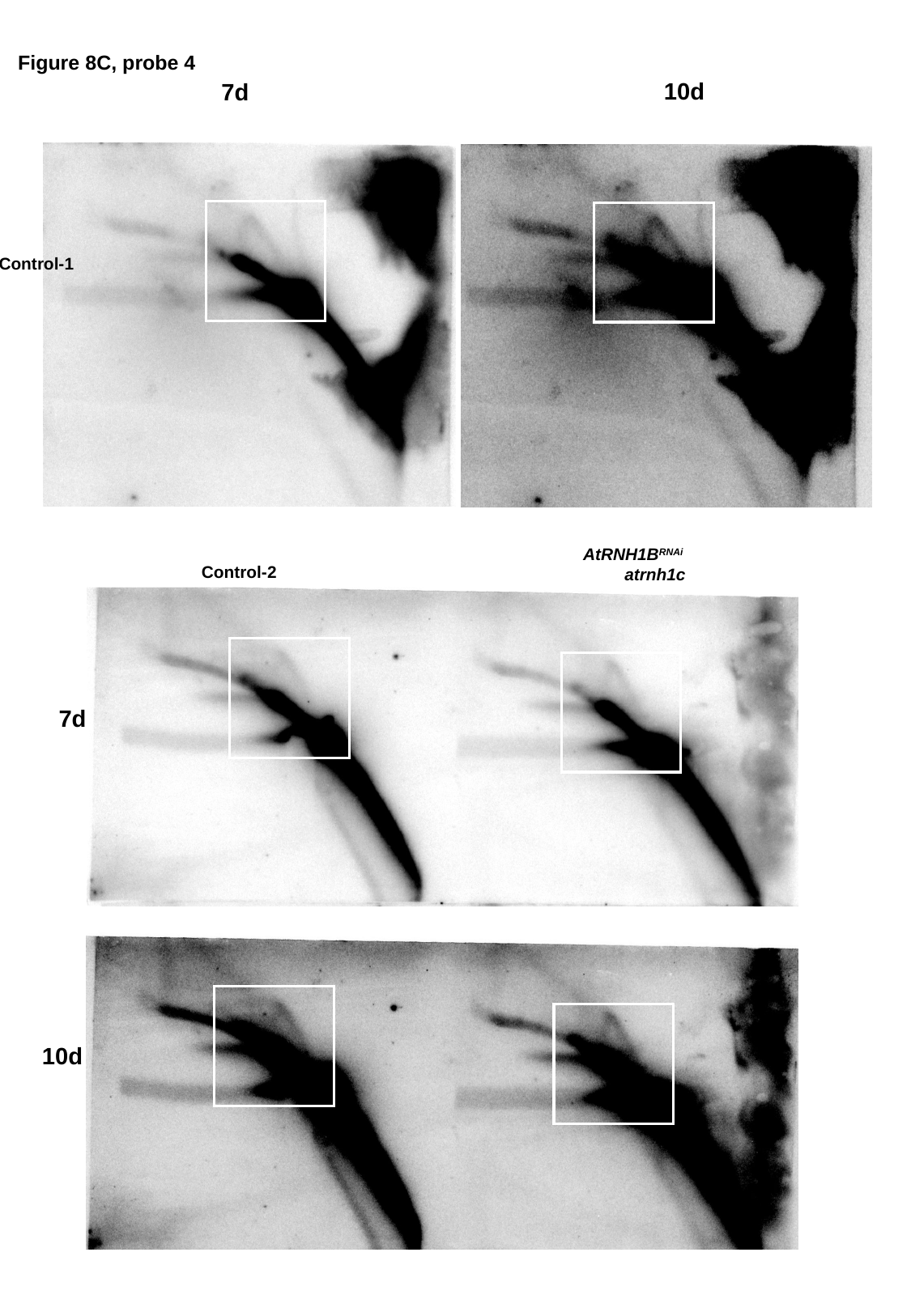

Figure 8C, probe 4
10d
7d
Control-1
AtRNH1BRNAi
atrnh1c
Control-2
7d
10d

## Slide 12
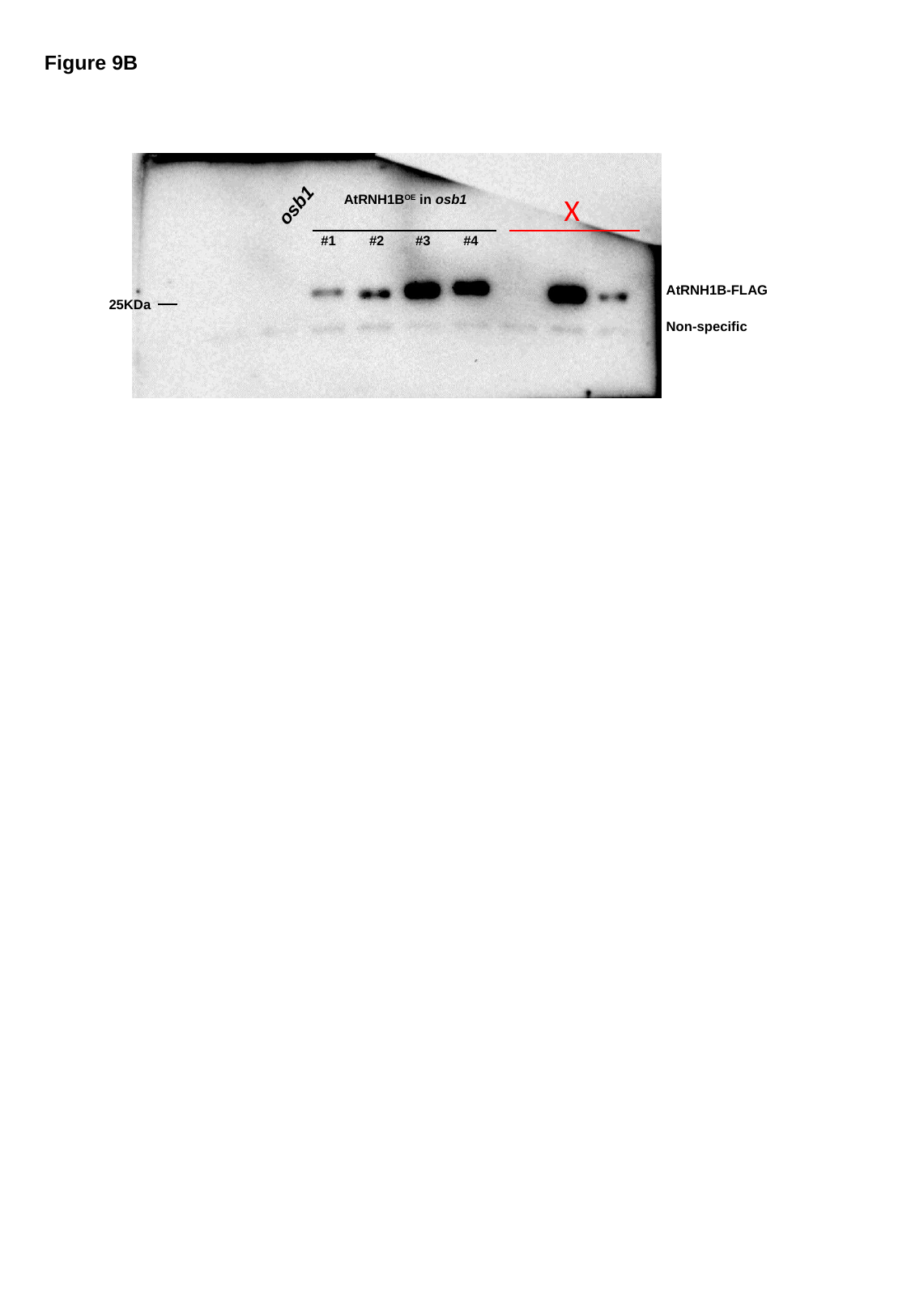

Figure 9B
osb1
AtRNH1BOE in osb1
#1
#2
#3
#4
X
AtRNH1B-FLAG
25KDa
Non-specific

## Slide 13
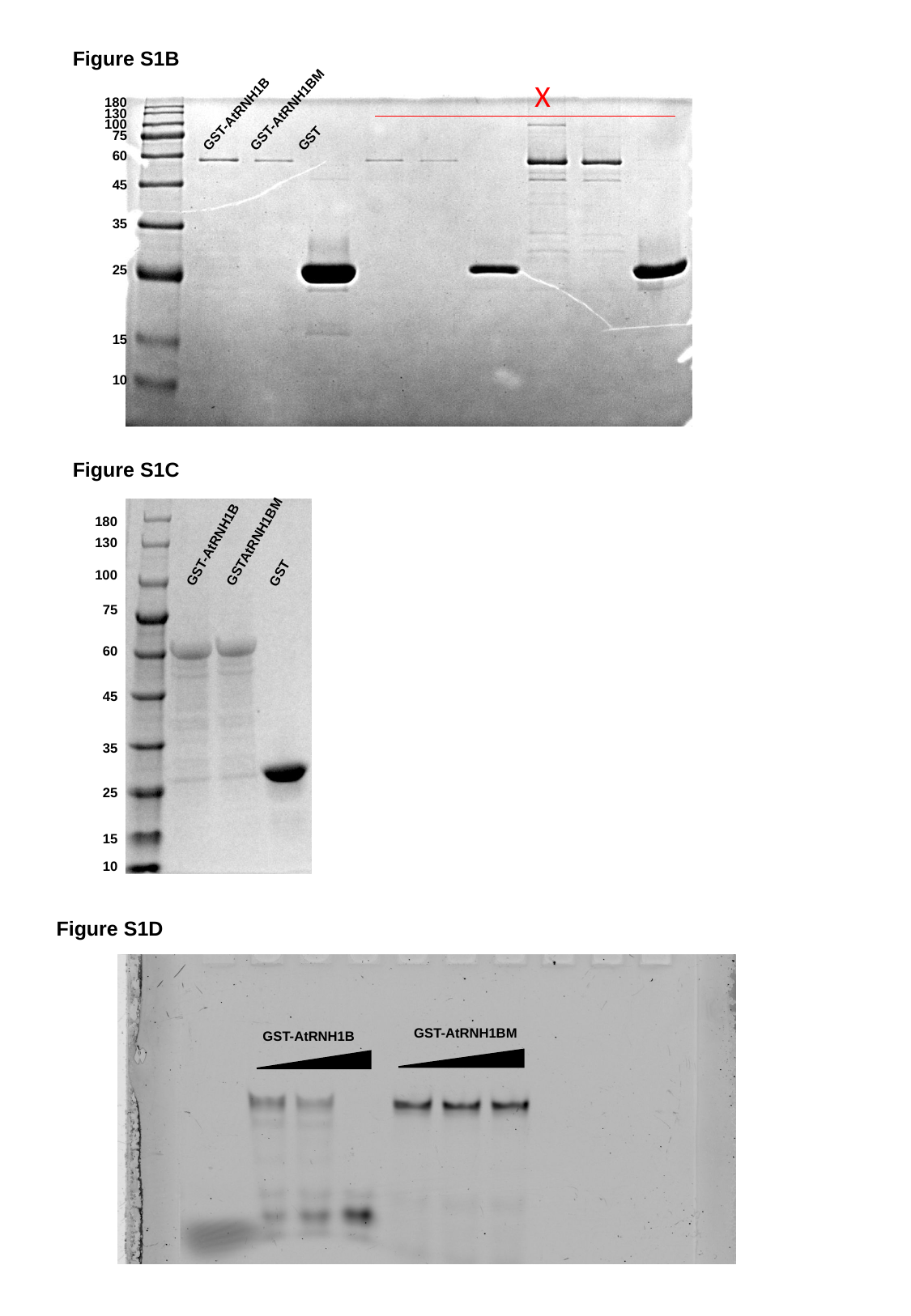

Figure S1B
X
180
GST-AtRNH1BM
130
GST-AtRNH1B
100
75
GST
60
45
35
25
15
10
Figure S1C
180
GSTAtRNH1BM
130
GST-AtRNH1B
GST
100
75
60
45
35
25
15
10
Figure S1D
GST-AtRNH1BM
GST-AtRNH1B

## Slide 14
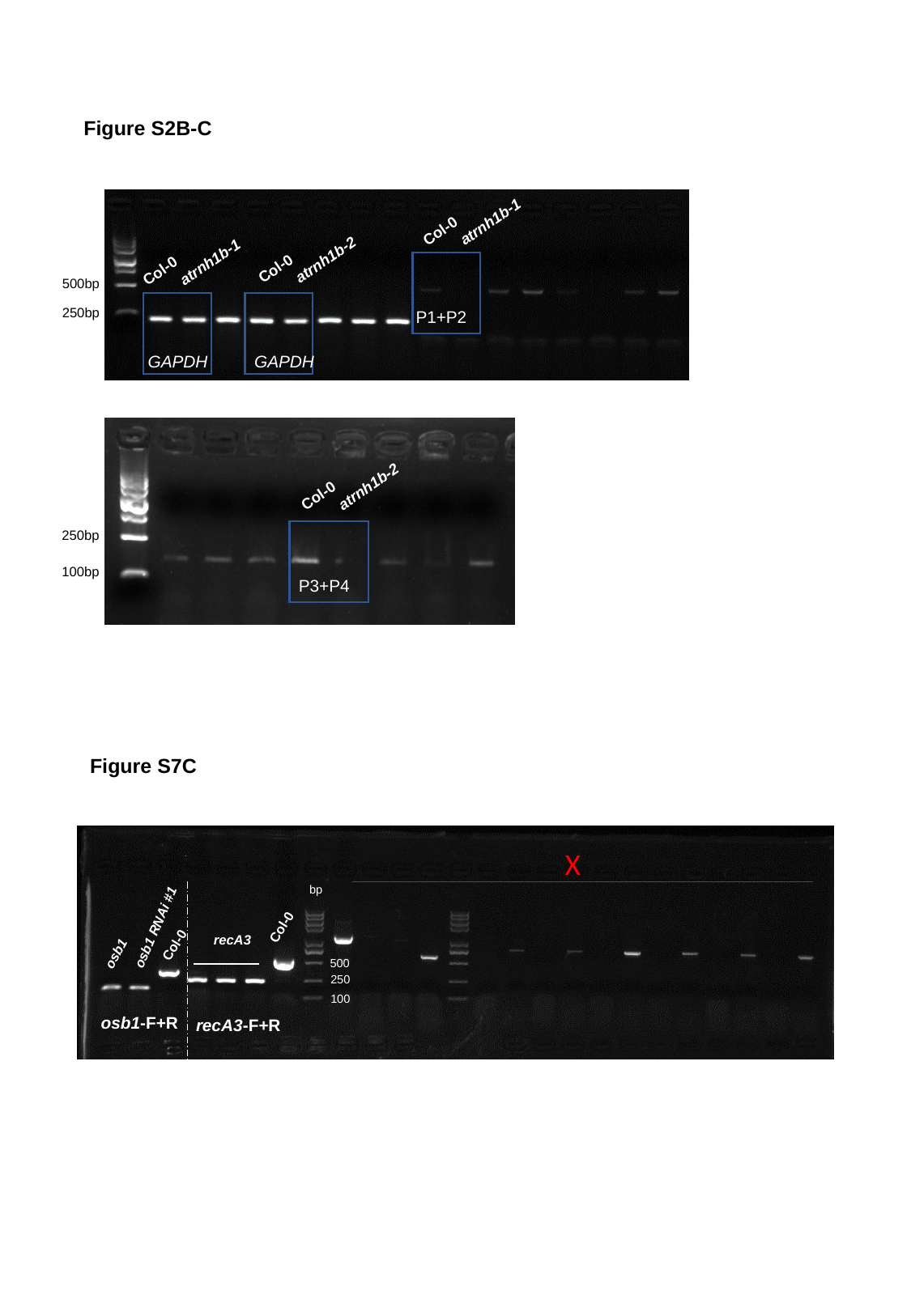

# Figure S2B-C
atrnh1b-1
Col-0
atrnh1b-2
atrnh1b-1
Col-0
Col-0
500bp
250bp
P1+P2
GAPDH
GAPDH
atrnh1b-2
Col-0
250bp
100bp
P3+P4
Figure S7C
X
bp
Col-0
osb1 RNAi #1
recA3
Col-0
osb1
500
250
100
osb1-F+R
recA3-F+R
