# Supplementary material for: Mitochondrial RNase H1 activity regulates R-loop homeostasis to maintain genome integrity and enable early embryogenesis in Arabidopsis
Source: PLoS Biol. 2021 Aug 3;19(8):e3001357. doi: 10.1371/journal.pbio.3001357 (PMC8330923; doi:10.1371/journal.pbio.3001357)
Supplement: Attachment — Submitted filename: Response-20210522.docx [file pbio.3001357.s013.docx]

We would like to thank all the reviewers for their constructive comments. We hope that they could find our answers satisfactory.

Reviewers’ comments

**Rev. 1:**

This is a well-written and thoroughly presented manuscript reporting on the role of RnaseH in maintaining organelle genome integrity in Arabidopsis, and it's expression levels and role in embryogenesis. The experiments and resulting data are clear and the presentation and discussion of the results are founded in the data. I found the work to be convincing, and this provides a very helpful addition to our knowledge of mechanisms involved in replication and maintenance organelle genomes during plant development. I have two minor points as follows.

1. In the introduction the size of plant mitochondrial genomes is mentioned. In recent years much larger mitochondrial genomes have been characterized in some angiosperms, particularly Silene. PNAS August 18, 2015 112 (33) 10185-10191; first published May 5, 2015; https://doi.org/10.1073/pnas.1421397112 This could be updated in your manuscript.

Response: Thanks very much for the information. We added the reference and corrected the size accordingly (Line 56).

2. Two-dimensional agarose gels can be quite difficult to evaluate. The ones you show have a high level of background, but I think they are sufficient to support the conclusions you make from them. These are some of the better ones I've seen in plant organelle manuscripts, so I don't have any major concern with these in this paper. I do not have any suggestions for anything different as this work adds an additional way to look at the plants.

Response: Thank you for the very positive appreciation of our work.

**Rev. 2:**
This is a very interesting study of the RNase H1 protein in plants that functions in both mitochondrial and plastid DNA stability. The investigators describe evidence of its role in mitochondrial genome stability and, in association, plant development. The study provides compelling evidence that the RNase H1 protein in mitochondrial participates with other components, most notably OSB, in its function, and influences homologous recombination within mitochondrial repeated sequences.

The manuscript is well written and informative. However, there are a few areas where clarification would help the reader to place this work in the context of what we currently know about plant mitochondrial and plastid genome behavior.  

1.     The authors carry out much of their protein targeting work in protoplasts that appear to represent leaf mesophyll cells. However, the developmental expression of the RNase H1 is not in mesophyll but in vascular tissue, meristem, reproductive/embryo and perhaps epidermis (the images appear to show expression in trichomes?). Therefore, one assumes that the process of mitochondrial DNA replication, presumably accompanying biogenesis, is likely enriched in meristem, reproductive tissue and embryo. It is not clear why there is enhanced signal in vascular tissues, but it is possible that this gene, like many others that dually target mitochondria and plastids, may play multiple roles. At very least it is important to be precise in the text to make clear that the mesophyll chloroplasts being tested for targeting are not organelles in which this protein likely resides.
Response: We agree with you that protoplasts mainly represent mesophyll cells and AtRNH1B is highly expressed in vascular tissue and epidermis (as shown by GUS staining in Figure 2A). However, we think AtRNH1B is also expressed in mesophyll cells, as we observed the AtRNH1B-GFP signal in the mesophyll cells in detached leaves of *AtRNH1Bpro*:*AtRNH1B*-*GFP atrnh1b* transgenic line (Figure 1A). More importantly, the GUS signals were also detected in the mesophyll cell of *AtRNH1Bpro:AtRNH1B-GUS atrnh1b-1* (Figure S1E and Movie S2).

We fully agree with the idea that AtRNH1B may play multiple roles, and in consistent with this idea, we found the shapes/patterns of mitochondria in different tissues are much different (respFigure 1A). Nevertheless, our confocal microscopy observation (Figure 1A and 5D), and organelle fractionation and immunoblot analysis (respFigure 1B, shown as Figure 1C in current version) determined that AtRNH1B is a mitochondria-specific rather than dual targeting protein.

**respFigure 1**. (**A**) Mitochondrial morphology in various tissues and cells of Col-0 Arabidopsis. Scale bars, 10 μm. Magenta= MitoTracker. (**B**) Localization of AtRNH1B protein by immunoblot analysis.

2.     I would argue that there is not a "canonical" dual targeting protein configuration in plants as suggested by the authors, or they should be specific in defining it. The authors indicate that there is an internal regulatory signal that defines plastid and mitochondrial targeting behavior in response to cellular signaling.  This appears to be a reasonable interpretation of the data. But this mechanism is likely not new, and the authors should have provided more information about the sequence located at 69-77 aa downstream from the translation start. Twin presequences have been described in many plant genes, and alternative translation initiation, including non-ATG start sites, have been demonstrated in a number of plant genes including those organellar targeted.  One straightforward interpretation of the results presented is that the "plastid targeting" form AtRNH1C is actually controlled by a twin presequence that permits alternative translation initiation, with thedomain 69-77 serving as the mitochondrial targeting domain.  Translation initiation at the predicted ATG start may compete with a second translation initiation site downsteam, providing the ability of a single gene to encode two forms of the product. This type of regulation has been reported for the organellar DNA polymerase (doi: 10.1105/tpc.108.063644) and would not be truly novel, but it is a very interesting feature and the amino acid sequence in the 69-77aa region, with its likely facilitation of mitochondrial targeting, should be addressed more clearly in this report.

Response: Thank you for the comments and information about twin presequence. We replaced “canonical” with “typical” (in line 402). In our study, we think the 69-77aa region functions as a blocker that prevents AtRNH1C from targeting mitochondria, because: 1) CTS region of AtRNH1C itself can deliver GFP into both chloroplasts and mitochondria (Figure 5A); 2) AtRNH1C△CTS-GFP failed to target into chloroplasts and mitochondria (Figure 5B and respFigure 2) and could not complement the *atrnh1c* mutant yellowish phenotype (see below respFigure 3 the results from Yang *et al*., The Plant Cell 2017); 3) Deletion of 69-77aa region confer the dual-localization of CTS^1C^-AtRNH1C-GFP (△69–77aa) in both transient transformation (Figure 5H) and stable transgenic plants (Figure 5I). The proposed alternative translation initiation skipping 69-77aa region would miss all the CTS signal sequence, which should abort the translocation of AtRNH1C into chloroplast and mitochondria.

We agree that we could not completely rule out the possibility that an alternative translation initiation results in a different organellar-specific signal sequence, as MTS and CTS are highly similar in general. However, in general, our results can fully support the model we have proposed; thus we keep our current model as it is. We are also very curious about the regulatory mechanisms behind, and through a forward genetic screen, we have found several mutants that have defects on the AtRNH1C translocation in *atrnh1b* mutant background. This ongoing work will uncover the regulatory mechanisms in the future.

**respFigure 2**. Results from Yang *et al*., The Plant Cell 2017. (**A**) AtRNH1C protein domain architecture. (**B**) Subcellular localization of GFP fused to the full-length AtRNH1C protein (upper panel) or to the AtRNH1C protein lacking the CTS (middle panel) and GFP alone (bottom). Bar = 10 μm.

3.     Is there evidence that the plastid form of the RNase H functions to stabilize the plastid genome, or functions within the plastid genome in the same manner as in the mitochondrion? Disruption of the mitochondrial gene is compensated by retargeting of the plastid form, but what is the consequence of disruption of the gene encoding the plastid form?

Response: In our previous studies, we had uncovered that the plastid RNase H1, AtRNH1C, participates in maintaining the integrity of plastid genome by resolving transcription-replication head-on conflict-triggered R-loops [1, 2]. We think, in both chloroplast and mitochondria, AtRNH1C functions as R-loops resolver in maintaining genome integrity, but we are not sure whether it works in the same manner, because the genome organization in plastid and mitochondria are not exactly the same, for example the HR surveillance mechanism is only discussed in mitochondria, and the transcription-replication triggered R-loops formation and resolution that we found in chloroplast [1, 2] are less understood in mitochondria. Different to the *atrnh1b* mutants, the *atrnh1c* mutant shows cpDNA damage and obvious yellowish phenotype, indicating that AtRNH1B can not compensate the loss of plastid RNase H in absence of AtRNH1C (respFigure 3 and Figure S5C), which is in consistent with its dominant mitochondria localization property (Figure 1A, 1C, and 7F).

**respFigure 3**. Results from Yang *et al*., The Plant Cell 2017. (**A**) The AtRNH1C gene and its encoded protein domains are shown. (**B**) RT-PCR analysis of AtRNH1C expression level in *atrnh1c*. (**C**) The phenotype of 20-d-old plants of Col-0, *atrnh1c*, CRISPR-mediated AtRNH1Cdeletion (*atrnh1c^del^*), and transgenic lines with full-length or truncations of AtRNH1C in *atrnh1c* mutant. (**D**) Rosette leaves of 2-week-old Col-0 (upper panel) and atrnh1c mutant (lower panel). Bar = 1 cm. (**E**) Photographs and chlorophyll fluorescence images of Col-0 (left) and atrnh1c (right)plants. (**F**) The chlorophyll contents in the leaves of 14-d-old Col-0 and *atrnh1c* plants. (**G**) and (**H**) Comparison of protoplasts from the leaves of 14-d-old Col-0 and *atrnh1c* plants. (**I**) PFGE of cpDNA obtained from the same (panel 1 from left) and different numbers (panel 3 from left) of chloroplasts from 2-week-old Col-0 and *atrnh1c* plants.

Minor issues:
In some of the figures using microscopy, arrows to indicate specific features like cristae would probably be helpful to general readers.
Response: Thank you for the suggestion. We added arrows and accompanying annotations (Figure 3E, 3F and S3E).

In Figure 3, it would be helpful to use more specific genetic description. What is the population size, what is the precise segregation?
Response: Thank you for the suggestion. The total aborted seeds/population sizes of the four genotypes are 13/513, 382/1458, 8/482 and 408/1628 seeds, respectively (Figure 3B). The information has been added in figure legend (Line 811-812).

**Rev. 3:**
In this manuscript, the authors studied the role of RNase H1 in the mitochondria in Arabidopsis thaliana. They argue that whilst AtRNH1B is the main RNase H1 isoform in the mitochondria, AtRNH1C can be targeted to the mitochondria when AtRNH1B is missing and compensate for its absence. A small motif in AtRNH1C prevents its targeting to the mitochondria in the presence of AtRNH1B but it is not clear how lack of AtRNH1B would relieve this self-inhibition. When both AtRNH1B&C are deficient, the plants are sterile, R-loops accumulate in the mitochondria and the frequency of HR events increases. The authors present evidence that RNH1 acts together with the ssDNA-binding protein Osb1 to control HR frequency but the mechanisms involved are unclear. Finally, the authors argue that, contrary to what happens in mammalian cells, R-loops do not participate in the replication of mitochondrial DNA but the evidence presented in support of this conclusion is rather weak. Whilst some of these observations are interesting, they fail to clarify the role of RNase H1 or R-loops in mitochondrial function or in the regulation of HR.

As they stand, these observations are a bit disparate and probably not of sufficient general interest. Perhaps this study would be better suited for a more specialized journal.

Response: We sincerely appreciate all your comments. In this study, we determined the subcellular localization and expression pattern of RNase H1 AtRNH1B. Moreover, we characterized its biological function in embryogenesis and uncovered the underlying regulation mechanism. The striking facultative dual-targeting mechanism between chloroplast and mitochondria have not been reported in any plant species, and this regulatory mechanism could be general in other organellar targeting proteins that have multiple localization capability from all organisms. In addition, our study had extended the role of R-loops in plant organellar genome stability. We agree with you that some details are missing at present, however, our research presents a relatively complete biological process and it would benefit researchers working on the areas of embryogenesis, organellar communications, nucleus signals, organellar protein importing, R-loops, homologous recombination, genome stability, who account for a large part of the broad readership of *PLOS Biology*.

Major points:   
-     Fig 1A: The distribution pattern of AtRNH1B-GFP and MitoTracker are very different and it is very difficult to conclude from these images that their infrequent colocalization does not simply happen by chance. At best, AtRNH1B-GFP would localize in a very small subset of mitochondria. The authors should (i) tone down their conclusion that AtRNH1B-GFP localizes to the mitochondria and (ii) speculate as to why they can only detect AtRNH1B-GFP in only a subset of mitochondria (what's special about those mitochondria?).
Response: We agree with you that it is hard to tell the subcellular localization of AtRNH1B as the MitoTracker signals were spread throughout the cell. To this point, we observed the GFP fluorescence in cells with mitochondria aggregated along the plasma membrane. As shown in respFigure 4 (now included in Figure 1A in current version), the GFP signals were mainly colocalized with MitoTracker signals. The Supplemental Movie 1 we had provided also showed that the GFP fluorescence moved together with MitoTracker signals. Moreover, the immunoblot analysis we include in this new version confirm the mitochondrial localization of AtRNH1B (respFigure 1B). Now, we think our data can fully support the conclusion that AtRNH1B localizes within mitochondria.

Detecting a mitochondria-localized protein in only a small part of the mitochondria by confocal observation had been observed [3]. We propose it may be because those mitochondria are highly activated and produce more R-loops that need more RNase H for resolution, which is consistent with the Supplemental Movie 1 that we had attached in previous version (see current version of Supplemental Movie 1, the AtRNH1B-GFP signals are overlapped with the higher speed of mitochondria movement). Moreover, what we had observed might just be because of uneven translocation of AtRNH1B in mitochondria.

**respFigure 4**. Confocal microscopy roots of *AtRNH1Bpro*:*AtRNH1B*-*GFP atrnh1b* transgenic plants.

-     Fig 1C: it is not clear how the authors have determined that the upper band corresponds to the unprocessed, MTS-containing AtRNH1B? How did they exclude that this faint band could correspond to another type of isoform (phospho-isoform for example)?
Response: We agree with you that we probably misinterpreted this band. Thus, we performed western blot analysis with purified organelles. We detected AtRNH1B both in total tissue and mitochondrial fractions but not in chloroplast (respFigure 1B, shown as Figure 1C in current version). We could not exclude that this faint band could correspond to another type of isoform (such as phospho-isoform), thus we had replaced to the current Figure 1C instead.

-     Fig 1D: to make the comparison meaningful, the authors should show clearly that the same amount of proteins has been put everywhere.

Response: Thank you for the suggestion, we added the Coomassie-stained membrane to indicate the protein amount we used (Figure S1C).

-     Fig 3A: AtRNH1Bpro: AtRNH1B△MTS-GFP atrnh1b 1c+/-; is there partial complementation here? The seeds look less damaged than in atrnh1b 1c+/-. Is it because the siliques have been photographed at an earlier stage of desiccation or is it that there is partial complementation? 
Response: We are sorry for the misleading information. As shown in Figure 3B and Figure S3C, AtRNH1B△MTS-GFP cannot complement the loss of AtRNH1B at all. The siliques we presented were indeed at different stages (see below respFigure 5). We have replaced the siliques in same developmental stage (Figure 3A).

**respFigure 5**. Dissected siliques of *atrnh1b 1c^+/-^* plant in different developmental stages. Asterisks indicate lethal seeds. Asterisks indicate lethal seeds. Bar = 200 μm.

-     Fig 4A: please provide the magnification for Col-0 to allow a proper comparison. 
Response: Thank you. The magnified image is now added in Figure 4A.

-     Lane 182: "the signals were exclusively localized in mitochondria". Same comment as for Fig 1A: this feels strongly like an over-statement.
Response: We agree with you that it is over-stated. We reformulated the sentence and toned down the statement (Line 183).

-     Lane 233: please explain exactly how the RNAi plants have been generated (detailed explanations in the methods section should be mandatory). 
Response: Thank you. The detailed explanations were added (Line 467-475).

-     Lane 242: To say that loss of mitochondrial RNase H1 triggers sterility is different from saying that it is important for embryogenesis. On Fig S5E, it looks as if the siliques of AtRNH1BRNAi atrnh1c have not been fertilized at all, hinting at a fertilization problem distinct from the problems of embryo development suggested on Figure 3C. Considering that the mature pollen expresses a lot of AtRNH1B (Figure 2B), could it be that the plants are male or female sterile? This is easy to test. It would also help to know how the plants have been pollinated (self-pollination?) and which cells express the RNAi construct: this should be made clear in the text (see above comment). 
Response: Thanks for the suggestions. The RNAi fragment was driven under 35S promoter; thus it is supposed to be expressed throughout the plants. But we only noticed the phenotype of silique defect (Figure S4D and S4E).

We had tested the pollen viability of *AtRNH1B^RNAi^ atrnh1c* by Alexander’s staining, which shows that the pollen of *AtRNH1B^RNAi^ atrnh1c* are indistinguishable from those of Col-0 (respFigure 6A, shown as Figure S5G in current version). Moreover, reciprocal crosses between Col-0 and *AtRNH1B^RNAi^ atrnh1c* and *atrnh1b 1c^+/-^* produced seeds normally (respFigure 6B and 6C, shown as Figure S3A and S5H in current version), indicating both the male and female gametophytes lacking RNase H are still fertile. It may be because the residual AtRNH1B in the *AtRNH1B^RNAi^ atrnh1c* #1 is enough for gametophyte, and we agree with the idea that AtRNH1B may function in fertilization.

**respFigure 6**. (**A**) Pollen viability in 5-week-old plants by Alexander staining. Scale bars, 10 μm. (**B**) and (**C**) Siliques from reciprocal crosses of Col-0 and RNAi #1 **(B)**, and Col-0 and *atrnh1b 1c*^+/-^ (**C**). Scale bars, 200 μm.

-     Fig 7A and Fig 9: please provide the raw data (% IP) of the DRIP-qPCR in order to give an idea of the actual strength of the DRIP signal. Please also provide at least one negative locus for comparison.

Response: In our previous study, we had found that the R-loops are produced all along mitochondrial DNA [4], thus we may not be able to provide a negative locus. Moreover, it is generally acceptable to include a RNase H pre-treated sample as negative control, without further providing negative locus, in DRIP-qPCR assay [5-7]. The raw data was provided in respFigure 8, 9, and 10.

**respFigure 7**. Snapshot of DRIP-seq signal in mitochondrial DNA of Col-0.

**respFigure 8**. Raw data related to Figure 7A.

**respFigure 9**. Raw data related to Figure 9A.

**respFigure 10**. Raw data related to Figure 10B.

-     Lane 272: "These results suggested that R-loops promote HR in Arabidopsis mitochondrial genome". Here the authors show that both R-loop and HR levels increase concomitantly but it is not enough to demonstrate that R-loops promote HR. It is only a correlation. This sentence is therefore an over-statement.
Response: Thanks for this comment. So far, many results had shown that RNA:DNA hybrids could promote HR-mediated DNA repair [8-12], including our recent study determined R-loops promote HR in chloroplast. Studies from [8, 9, 12] and our results in chloroplast (NAR accepted, attached as the support materials in this revision) all shown that the RNA:DNA hybrids promoting HR requires the similar modules including RNase H1 and ssDNA-binding proteins. This mechanism seems to be quite conserved among organisms, thus promoted us to draw the similar conclusion in this study. However, we agree that we could not applied the same mechanism into the case that in mitochondria, as lacking enough data to support this model. Thus, we downplayed the statements (Line 287).

-     Figure 7DE: The authors should investigate using Mitotracker whether there is an overall reduction in the number of mitochondria in atrnh1b/c. Could these data be consistent with a problem of mitochondria replication? 
Response: Thank you for the suggestion. We examined the mitochondria, and the MitoTracker signals in *atrnh1b/c* was indeed reduced (respFigure 11). Thus, the reduced copy number of mtDNA in single cell could be because of the reduction in the number of mitochondria, and/or the reduction in the copy number of mtDNA in single mitochondria. Due to the limitation of technology in single cell and single mitochondria, it is hard to make a thorough conclusion about the origin of the mitochondria replication problem.

**respFigure 11**. An overall reduction of mitochondria (labeled with the MitoTracker Orange dye) quantity in *atrnh1b/c* aborted seeds compared to Col-0 and atrnb1b-1 normal seeds. Magenta= MitoTracker. Scale bars, 10 μm.

-     Fig S6B/C: A statistical analysis of the significance of the data should be performed. 
Response: The statistical analysis was performed using One-way ANOVA, and the results were shown on the graph. The information had been added in the legend.

-     The results presented on Fig 8 are not very convincing. It is not clear how many times they have been performed. Quantifications should be provided to confirm the conclusions put forward by the authors.
Response: Thank you. We think the 2D-AGE assay is a relatively direct and precise method in determining mtDNA replication, while it is quite difficult to be conducted. After searching all the literatures, we could hardly find the example for quantifying results from 2D-AGE assay. To our knowledge, there are very few reports adapted this method to examine plant mtDNA replication and our data are much better [13, 14]. In this study, we intended to test the possibility whether the replication in plant mitochondria was inhibited in absence of RNase H as what happened in animal, thus we checked four loci through four times of experiment. All the results are in consistent at least in excluding this possibility, which makes the conclusion reliable.
-     Fig 9A: To validate the supposed increase in R-loop formation in osb1, it is essential to show that the increase in DRIP signal is actually sensitive to the addition of exogenous RNase H. It is unclear why the authors did not look at the same sites in Fig 7A and in Fig 9A. The putative increase in R-loop levels in the absence of Osb1 should be directly compared to the increase observed in the absence of mitochondrial RNase H1.

Response: Thanks for the suggestion. The DNA of *osb1* was pre-treated with RNase H and used for DRIP-qPCR. The result was added in Figure 9A (Please also see our raw data in respFigure 9).

According to the 2D-AGE results, we found the I region was more obviously affected, while the T region was unchanged. Thus, we focused on I region in following work. We think this alteration did not affect our conclusion.

Due to the lethality problem, we could not get enough DNA from *atrnh1b/c* mutant for DRIP-qPCR (same issue to 2D-AGE assay). Thus, we had used *AtRNH1B^RNAi #1^atrnh1c* for all DRIP-qPCR assay, and we had compared the R-loops in *osb1* with those in *AtRNH1B^RNAi #1^atrnh1c* (Figure 10B).

-     Fig 9D: The asymmetry of the HR reaction at L and I (L1/2 versus L2/1; I1/2 versus I2/1) is exactly the opposite in osb1 and in atrnh1b/c (see Fig 7C); and whilst the impact of atrnh1b/c was "symmetrical" at EE, it is strongly asymmetric in osb1. In addition, the impact of osb1 is much greater (*600) at EE2/1 than in atrnh1b/c (*30) and only partially compensated by the over-expression of AtRNH1B. At I, osb1 significantly impacts HR frequency whilst the authors did not detect an accumulation of R-loops. Taken together these observations strongly suggest that Osb1 and AtRNH1B/C do not work in the same pathway to control HR and that at the very least, a large part of the contribution of Osb1 to HR control is R-loop independent. The authors should therefore tone down their conclusions.

Response: We agree with you that the accumulation patterns of crossover products between *osb1* and *atrnh1b*/*c* were different. In fact, we had mentioned this in text (line 352-353). We had not highlighted Osb1 and AtRNH1B/C work in the same pathway. We proposed that AtRNH1B/C work synergistically with Osb1 in EE and I regions, and might work in the same pathway at L region. It may be because the model we provided caused misunderstanding. We had modified the model, and further tone down the conclusion (line 335).

-     Fig 10B: is there a mistake in the legend here? The authors say: (lane 328) "AtRNH1BRNAi atrnh1c showed an epistatic effect to osb1 at repeat L and an additive effect at repeat EE", whilst the DRIP signal in osb1 RNAi #1 (blue) is systematically much lower than in either single mutant. The conclusion clearly does not match the data. 
Response: Thanks for pointing out the mistake, indeed the results of *osb1* and *osb1 RNAi #1* were mixed up, as you can tell from Figure 9A that the R-loop ratios of *osb1* to Col-0 at repeat L and EE2 are about 3. The corrected graph is presented and the raw data is provided (respFigure 10).

Minor points:
-     Fig 1A: to facilitate the reading of the figure, it would be better not to include the Bright field in the merge. 
Response: Thanks for the suggestion, the bright field was removed.

-     Fig S1A: The pictures are incredibly pixelated and are not of sufficient quality to be published in their current state. Explain also to the non-specialists that chloroplasts can be observed in the bright field channel as round objects. 
Response: The Figure S1A was deleted, as we think the results shown in Figure 1A and 1C are sufficient to support the conclusion of the mitochondrial localization property of AtRNH1B.

-     Fig S1B: please explain the meaning of the different colors/domains (catalytic, HBD).
Response: The annotations of the domains were added in the schematic diagram.

-     Lane 154: "As the mitochondrial localization of AtRNH1B is responsible for embryo lethality". Should it not be: "As lack of the mitochondrial localization…" ?
Response: The sentence was reformulated to “As lack of the mitochondrial-localized RNase H is responsible for embryo lethality” (line 156).

-     Lane 274: "Because frequent ectopic HR is a source of genome integrity". Presumably, replace "integrity" by "instability". 
Response: Thanks for the suggestion. We have corrected this (line 289).

-     Fig 7F would probably benefit from being moved to Fig 1 to confirm the mitochondrial localization of AtRNH1B. Similarly, Fig 7G should be moved to Fig 4 to validate the mitochondrial localization of AtRNH1C in the absence of AtRNH1B. 
Response: Thanks for the suggestion. In this new version, we performed western blot analysis with purified mitochondria and chloroplasts, which confirmed the subcellular localization of AtRNH1B (Figure 1C, respFigure 1B) and AtRNH1C (Figure 6D, respFigure 12). Thus, we think it may be more suitable to keep the original style of Figure 7.

**respFigure 12**. Characterize the localization of AtRNH1C protein by immunoblot. Intact chloroplasts and mitochondria were isolated from three-week-old AtRNH1Cpro:AtRNH1C-GUS atrnh1c and AtRNH1Cpro:AtRNH1C-GUS atrnh1b transgenic plants. Anti-GUS polyclonal antibody was used to detect the AtRNH1C-GUS, and polyclonal antibodies anti-psbO, anti-IDN1, and anti-H3 were used to indicate chloroplast, mitochondria, and total protein fractions, respectively. Total, total proteins from leaves; Chl, proteins from isolated chloroplasts; Mito, proteins from isolated mitochondria.

-     Lane 313: "we assessed mitochondrial R-loops in msh1, osb1, and recA3 by DRIP-qPCR (Figure S7)". No DRIP experiments are displayed on Fig S7.

Response: Figure S7 contains the genotypic information about the mutants we tested, but we are aware that it was not put in the right position. Now we adjusted the position (Line 328). DRIP-qPCR results were shown in Figure 9A (Line 330).

**References**

1.	Yang Z, Li M, Sun Q: **RHON1 Co-transcriptionally Resolves R-Loops for Arabidopsis Chloroplast Genome Maintenance**. *Cell Rep* 2020, **30**(1):243-256 e245.

2.	Yang Z, Hou Q, Cheng L, Xu W, Hong Y, Li S, Sun Q: **RNase H1 Cooperates with DNA Gyrases to Restrict R-Loops and Maintain Genome Integrity in Arabidopsis Chloroplasts**. *Plant Cell* 2017, **29**(10):2478-2497.

3.	Guan X, Okazaki Y, Zhang R, Saito K, Nikolau BJ: **Dual-Localized Enzymatic Components Constitute the Fatty Acid Synthase Systems in Mitochondria and Plastids**. *Plant Physiol* 2020, **183**(2):517-529.

4.	Xu W, Li K, Li S, Hou Q, Zhang Y, Liu K, Sun Q: **The R-loop Atlas of Arabidopsis Development and Responses to Environmental Stimuli**. *Plant Cell* 2020.

5.	Perez-Calero C, Bayona-Feliu A, Xue X, Barroso SI, Munoz S, Gonzalez-Basallote VM, Sung P, Aguilera A: **UAP56/DDX39B is a major cotranscriptional RNA-DNA helicase that unwinds harmful R loops genome-wide**. *Genes Dev* 2020, **34**(13-14):898-912.

6.	Feretzaki M, Pospisilova M, Valador Fernandes R, Lunardi T, Krejci L, Lingner J: **RAD51-dependent recruitment of TERRA lncRNA to telomeres through R-loops**. *Nature* 2020.

7.	Graf M, Bonetti D, Lockhart A, Serhal K, Kellner V, Maicher A, Jolivet P, Teixeira MT, Luke B: **Telomere Length Determines TERRA and R-Loop Regulation through the Cell Cycle**. *Cell* 2017, **170**(1):72-85 e14.

8.	Liu S, Hua Y, Wang J, Li L, Yuan J, Zhang B, Wang Z, Ji J, Kong D: **RNA polymerase III is required for the repair of DNA double-strand breaks by homologous recombination**. *Cell* 2021, **184**(5):1314-1329 e1310.

9.	Ohle C, Tesorero R, Schermann G, Dobrev N, Sinning I, Fischer T: **Transient RNA-DNA Hybrids Are Required for Efficient Double-Strand Break Repair**. *Cell* 2016, **167**(4):1001-1013 e1007.

10.	Yasuhara T, Kato R, Hagiwara Y, Shiotani B, Yamauchi M, Nakada S, Shibata A, Miyagawa K: **Human Rad52 Promotes XPG-Mediated R-loop Processing to Initiate Transcription-Associated Homologous Recombination Repair**. *Cell* 2018, **175**(2):558-570 e511.

11.	Ouyang J, Yadav T, Zhang J-M, Yang H, Rheinbay E, Guo H, Haber DA, Lan L, Zou L: **RNA transcripts stimulate homologous recombination by forming DR-loops**. *Nature* 2021.

12.	D'Alessandro G, Whelan DR, Howard SM, Vitelli V, Renaudin X, Adamowicz M, Iannelli F, Jones-Weinert CW, Lee M, Matti V *et al*: **BRCA2 controls DNA:RNA hybrid level at DSBs by mediating RNase H2 recruitment**. *Nat Commun* 2018, **9**(1):5376.

13.	Manchekar M, Scissum-Gunn K, Song D, Khazi F, McLean SL, Nielsen BL: **DNA recombination activity in soybean mitochondria**. *J Mol Biol* 2006, **356**(2):288-299.

14.	Manchekar M, Scissum-Gunn KD, Hammett LA, Backert S, Nielsen BL: **Mitochondrial DNA recombination in Brassica campestris**. *Plant Science* 2009, **177**(6):629-635.
