# Supplementary material for: Mitochondrial RNase H1 activity regulates R-loop homeostasis to maintain genome integrity and enable early embryogenesis in Arabidopsis
Source: PLoS Biol. 2021 Aug 3;19(8):e3001357. doi: 10.1371/journal.pbio.3001357 (PMC8330923; doi:10.1371/journal.pbio.3001357)
Supplement: Attachment — Submitted filename: Response to reviewers.docx [file pbio.3001357.s014.docx]

Reviewers' comments

Reviewer #2:
I believe that the reviewers have satisfactorily addressed the concerns expressed in the previous review of this manuscript.
--Response: Thank you for the recognition of our work.


Reviewer #3:

The authors have made substantial corrections to their manuscript and its quality has improved as a result. I still think that this story would fit better in a more specialized journal and that the role of RNase H1 in the maintenance and replication of Arabidopsis mitochondria is still not entirely clear, as admitted by the authors themselves.
--Response: Thanks for the suggestion. As we had mentioned previously, we believe *PLOS Biology* is the home to original research with a broad scope across the biological sciences, and our research is indeed within this scope. In general, it is very difficult to clear up all the related questions in one single paper, but we think, at the moment, we had answered almost all key questions related to this manuscript.

I suggest that the raw DRIP data should be shown in the supplementary material.

--Response: Thanks for the suggestion. All the raw data and images can be found in S1 Data and S1 Raw Images files, respectively.

I suggest that the respFigure 11 should be added to the manuscript to balance the conclusions that RNase H1 does not play a role in the replication of mitochondria in Arabidopsis.
--Response: Thanks for the suggestion. We added this figure to the manuscript (shown as S3F Fig in the current version), and we added some statements about it (Line 160- 161, and Line 385-386).

Corrections to the text:
L226: replace "increasement" by "increase".

--Response: Thanks for point out the misnomer, we have corrected it.

L228: "there are much more immunogold labeled GFP particles detected in atrnh1b chloroplast than that in atrnh1b chloroplast" should be "than in atrnh1c chloroplasts".

--Response: Thanks for pointing out the mistake, we have corrected it.
